# Structural mechanism of TRPM7 channel regulation by intracellular magnesium

**DOI:** 10.1007/s00018-022-04192-7

**Published:** 2022-04-07

**Authors:** Eva Schmidt, Chamali Narangoda, Wolfgang Nörenberg, Miyuki Egawa, Anna Rössig, Marion Leonhardt, Michael Schaefer, Susanna Zierler, Maria G. Kurnikova, Thomas Gudermann, Vladimir Chubanov

**Affiliations:** 1 Walther-Straub Institute of Pharmacology and Toxicology, LMU Munich, Munich, Germany; 2 Chemistry Department, Carnegie Mellon University, Pittsburgh, PA USA; 3 Rudolf-Boehm Institute of Pharmacology and Toxicology, Leipzig University, Leipzig, Germany; 4 Institute of Pharmacology, Johannes Kepler University Linz, Linz, Austria; 5 Comprehensive Pneumology Center, a member of the German Center for Lung Research (DZL), Munich, Germany

**Keywords:** TRPM7, TRP channels, Magnesium, PIP_2_, ATP, Molecular dynamics simulations

## Abstract

Zn^2+^, Mg^2+^ and Ca^2+^ are essential divalent cations implicated in many metabolic processes and signalling pathways. An emerging new paradigm is that the organismal balance of these cations predominantly depends on a common gatekeeper, the channel-kinase TRPM7. Despite extensive electrophysiological studies and recent cryo-EM analysis, an open question is how the channel activity of TRPM7 is activated. Here, we performed site-directed mutagenesis of mouse TRPM7 in conjunction with patch-clamp assessment of whole-cell and single-channel activity and molecular dynamics (MD) simulations to show that the side chains of conserved N1097 form an inter-subunit Mg^2+^ regulatory site located in the lower channel gate of TRPM7. Our results suggest that intracellular Mg^2+^ binds to this site and stabilizes the TRPM7 channel in the closed state, whereas the removal of Mg^2+^ favours the opening of TRPM7. Hence, our study identifies the structural underpinnings through which the TRPM7 channel is controlled by cytosolic Mg^2+^, representing a new structure–function relationship not yet explored among TRPM channels.

**Supplementary Information:**

The online version contains supplementary material available at 10.1007/s00018-022-04192-7.

## Introduction

The transient receptor potential cation channel, subfamily M, member 7 (TRPM7) encodes a bi-functional protein comprising a transmembrane channel segment fused to a serine/threonine-protein kinase domain [1–3]. The TRPM7 channel is highly permeable to divalent cations, including Zn^2+^, Ca^2+^, and Mg^2+^, and there is mounting evidence to suggest that the influx of all three cations underlies the indispensable physiological role of TRPM7 [4–8]. Thus, TRPM7 controls a wide range of biological processes such as organismal Zn^2+^, Ca^2+^ and Mg^2+^ homeostasis, embryonic development, immune responses, cell motility, proliferation and differentiation [1–3].

TRPM7 currents were discovered in patch-clamp experiments with immune cells after removing Mg^2+^ from pipette solutions [9–11]. In contrast, the addition of free Mg^2+^ and Mg·ATP prevented the opening of the TRPM7 channel through different mechanisms [11–13]. Moreover, free Mg^2+^ and Mg·ATP inhibit TRPM7 synergistically, since elevation of free Mg^2+^ concentrations increases the potency of Mg·ATP [13]. TRPM7 currents were thus termed magnesium-nucleotide-regulated metal ion currents (MagNuM) and magnesium-inhibited cation currents (MIC) [11, 12]. In the past two decades, this experimental paradigm was commonly used to identify TRPM7 currents in a wide variety of primary isolated cells and stable cell lines [1–3]. The current consensus is that both Mg^2+^ and Mg·ATP act as negative regulators of the ubiquitously expressed TRPM7 channel to correlate the uptake of essential metals with the metabolic state of the cell [1–3].

The molecular mechanism determining TRPM7 sensitivity to intracellular Mg^2+^ remains unclear. Previously, several research groups examined the role of the C-terminal kinase domain with regard to TRPM7 channel sensitivity to Mg^2+^ and obtained controversial results. Thus, deletion of the kinase domain resulted in either non-functional versions of the channel [14], channel variants with unchanged [15] or even increased sensitivity to free Mg^2+^ [5, 15, 16]. In other studies, the introduction of ‘kinase-dead’ point mutations (D1775A and K1648R) in the catalytic site of the kinase moiety or deletion of phosphorylation residues (S1511 and S1567) did not affect the responses of the channel to free Mg^2+^ [14, 17, 18]. However, other researchers observed that TRPM7 with ‘kinase-dead’ mutations (K1648R and G1799D) exhibited reduced responses to free Mg^2+^ [5, 13], whereas the T1482L mutation affecting a putative phosphorylation site in TRPM7 increased Mg^2+^ sensitivity of the channel [19]. While the reason for such inconsistencies remains unclear, the overall consensus is that functional TRPM7 channel variants with impaired kinase moiety retain the sensitivity to intracellular free Mg^2+^, arguing that an additional Mg^2+^ regulatory domain must exist.

Other studies suggested that Mg^2+^ affects the TRPM7 channel indirectly, for instance, by interfering with the interaction of phosphatidylinositol 4,5-bisphosphate (PIP_2_) and TRPM7, but the structural basis of such interference remains unknown [20–22]. More recently, high-resolution structures of a closed TRPM7 channel were resolved by cryo*-*electron microscopy (cryo-EM) [23]. However, structural rearrangements associated with the Mg^2+^- and PIP_2_-dependent opening of the TRPM7 channel were not identified in the structures available [23].

Here, we embark on extensive functional analysis, structural modelling and molecular dynamics simulations and propose that a pivotal Mg^2+^ regulatory site of TRPM7 is located within the lower channel gate. Our findings suggest that Mg^2+^ interacts directly with this protein segment and stabilizes TRPM7 in the closed state. In line with this model, we found that a point mutation introduced in the Mg^2+^ regulatory site abolishes the sensitivity of TRPM7 to physiological concentrations of intracellular Mg^2+^.

## Results

### Search for amino acid residues involved in the Mg^2+^-induced inhibition of TRPM7

**Fig. 1:**
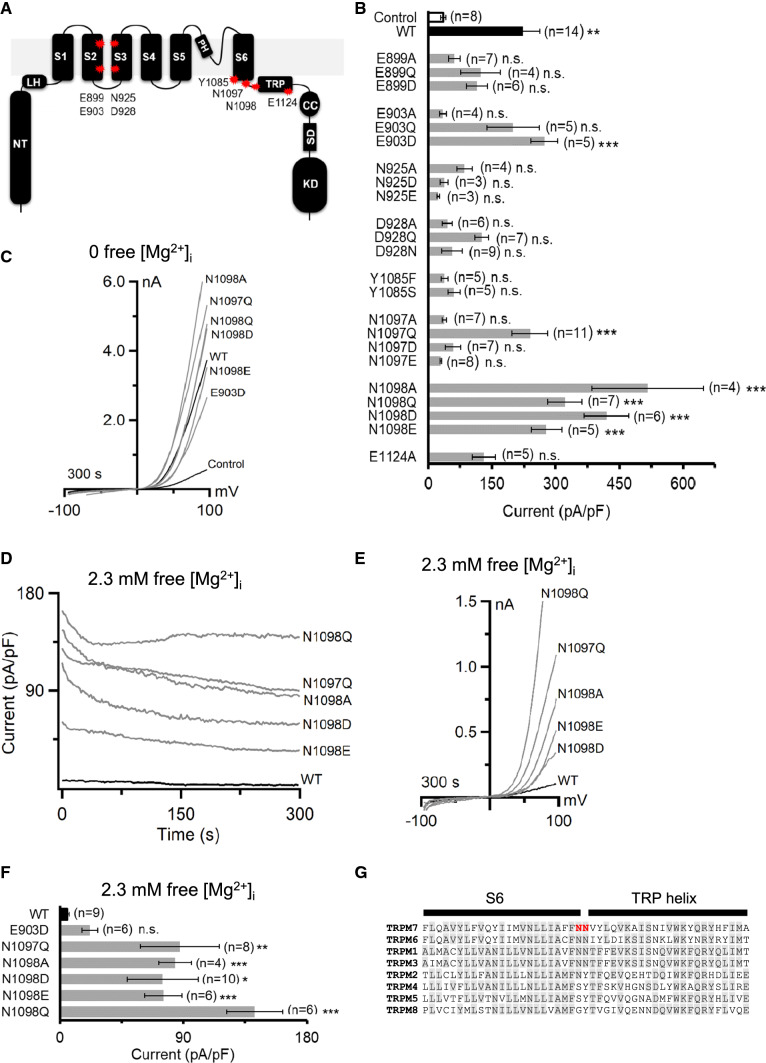
Assessment of the mouse TRPM7 variants expressed in HEK293T cells. **A** Domain topology of TRPM7. A large N-terminus (*NT*) of TRPM7 is linked to a pre-S1 helix also known as a linker-helical domain (*LH*) preceding a channel segment comprising six transmembrane helices (*S1–6*) with a short pore loop and a pore helix (*PH*) located between S5 and S6. The C-terminus of TRPM7 contains a receptor potential domain (*TRP*) followed by a coiled-coil (*CC*), kinase substrate (*SD*) and kinase (*KD*) domains. Red stars indicate the position of residues in the mouse TRPM7 protein subjected to site-directed mutagenesis in the present study. **B** Whole-cell currents measured in untransfected HEK293T cells (Control, white) and the cells transfected by wild type (WT, black) or indicated mutant variants (grey) of TRPM7-YFP cDNAs. Currents were induced using the standard [Mg^2+^]_i_-free intracellular solution and the standard external solution. Current amplitudes (mean ± SEM) were acquired at + 80 mV (at 300 s). *n*, number of cells measured; n.s., not significant; *P< 0.05 **P< 0.01; ***P< 0.001 (ANOVA, compared to Control). **C** Representative I–V relationships of currents shown in **B**. **D**–**F** A subset of TRPM7-YFP variants shown in **B** were examined using an intracellular solution containing 2.3 mM [Mg^2+^]_i_ (Suppl. Table S1). **D** Whole-cell currents measured in WT and indicated mutant variants of TRPM7-YFP. Current amplitudes (mean ± SEM) were acquired at − 80 and + 80 mV and plotted over time. **E** Representative I–V relationships of currents (at 300 s) illustrated in (**D**). **F** Bar graphs of outward currents (mean ± SEM; + 80 mV) shown in (D) at 300 s. *n*, number of cells measured; n.s., not significant; *P< 0.05 **P < 0.01; ***P <0.001 (ANOVA, compared to WT). **G** Multiple sequence alignment (ClustalW) of amino acid sequences encoding the S6 and TRP segments in the mouse TRPM1 – 8 proteins. Grey background indicates the sequence consensus. N1097 and N1098 of TRPM7 are indicated in red

Recently, several structures of TRPM channels were determined using cryo-EM [24–33]. The investigators noted that TRPM2, TRPM4, TRPM5 and TRPM8 harbour intra-subunit Ca^2+^-binding sites formed by five negatively charged and polar residues in the S2 and S3 helices and the TRP segment (Suppl. Fig. S1) [24–33]. Interestingly, some of these residues are substituted in TRPM1, TRPM3, TRPM6 and TRPM7 (Suppl. Fig. S1), suggesting that the latter group of channels may contain a binding pocket for another ligand, for instance, Mg^2+^. To test this hypothesis functionally, we exchanged E899, E903, N925, D928 and E1124 of TRPM7 to an uncharged alanine residue (A) or two structurally related acidic (D or E) and two polar residues (Q or N) (Fig. 1A).

In the pore domain formed by the S5 and S6 helices, the polar side chains of N1097 and N1098 appear to arrange the narrowest constriction in the lower gate of TRPM7 [23]. Since asparagine side chains frequently contribute to Mg^2+^-binding sites in other channels [34, 35], we hypothesized that N1097 and N1098 could function as an Mg^2+^ recognition site in TRPM7. Accordingly, we produced versions of TRPM7 with modified amino acids at positions N1097 and N1098 (Fig. 1A). Finally, we introduced changes in Y1085 (Fig. 1A) since its side chain hydroxyl group is located in the upper channel gate and may potentially interact with Mg^2+^ [23]. Because of the exceptionally low efficiency of in vitro site-directed mutagenesis of untagged mouse TRPM7 cDNA in the bicistronic pIRES2-EGFP vector, the functional impact of the introduced mutations was investigated using mouse TRPM7 with a C-terminal YFP tag in the pcDNA3.1 vector (see further details in “Materials and methods”).

First, we studied whether the TRPM7 mutants can be activated in the absence of cytosolic Mg^2+^, assuming that mutations with a specific impact on Mg^2+^-dependent inhibition of the channel should not significantly affect current amplitudes and current–voltage (I–V) relationships of TRPM7 in such an experimental setting. To this end, we transiently transfected HEK293T cells with cDNAs encoding wild-type (WT) and mutant versions of TRPM7-YFP and conducted patch-clamp experiments with YFP-positive cells. Whole-cell currents were elicited by a voltage ramp protocol ranging from − 100 to + 100 mV and a standard Mg^2+^-free internal solution (Fig. 1B, C). I–V relationships of WT currents exhibited characteristic features, such as tiny inward and large outward currents with a pronounced rectification and a reversal potential of about 0 mV (Fig. 1C). Accordingly, outward currents of TRPM7 variants were used to reliably quantify the effects of the mutations (Fig. 1B). Only six mutant variants (E903D, N1097Q, N1098A, N1098Q, N1098D and N1098E) displayed currents significantly different from endogenous currents in untransfected cells (Fig. 1B). In addition, I–V relationships of these mutant channels resembled those of WT currents (Fig. 1C). Consequently, only the latter six TRPM7 variants were selected for further analysis.

Next, we examined the channel activity of WT and mutant TRPM7 variants in the presence of relatively high levels of intracellular Mg^2+^.  As expected, the addition of free 2.3 mM [Mg^2+^]_i_ to the internal solution completely prevented the development of WT currents (Fig. 1D, F). Five channel variants (N1097Q, N1098A, N1098Q, N1098D and N1098E) displayed currents already after break-in, which were modestly reduced over time and exhibited typical I–V characteristics (Fig. 1F, D). In contrast, the E903D variant showed low activity under these experimental conditions (Fig. 1F). Hence, unlike other mutations, exchanges of asparagine residues located in the S6 segment and the TRP helix (Fig. 1G) resulted in active TRPM7 channels in the presence of [Mg^2+^]_i_. Consequently, we selected the N1097Q and N1098Q variants for a more detailed assessment.

### Impact of N1097Q and N1098Q mutations on TRPM7 channel inhibition by Mg^2+^and Ba^2+^

To rule out a potential influence of the YFP tag on the functional analysis of TRPM7, we re-introduced the N1097Q and N1098Q amino acid exchanges into mouse TRPM7 cDNA inserted into the bicistronic pIRES2-EGFP vector [36, 37]. Immunofluorescent staining of HEK293T cells expressing WT and mutant versions of TRPM7 did not reveal differences in the subcellular distribution of the proteins (Suppl. Fig. S2).

**Fig. 2:**
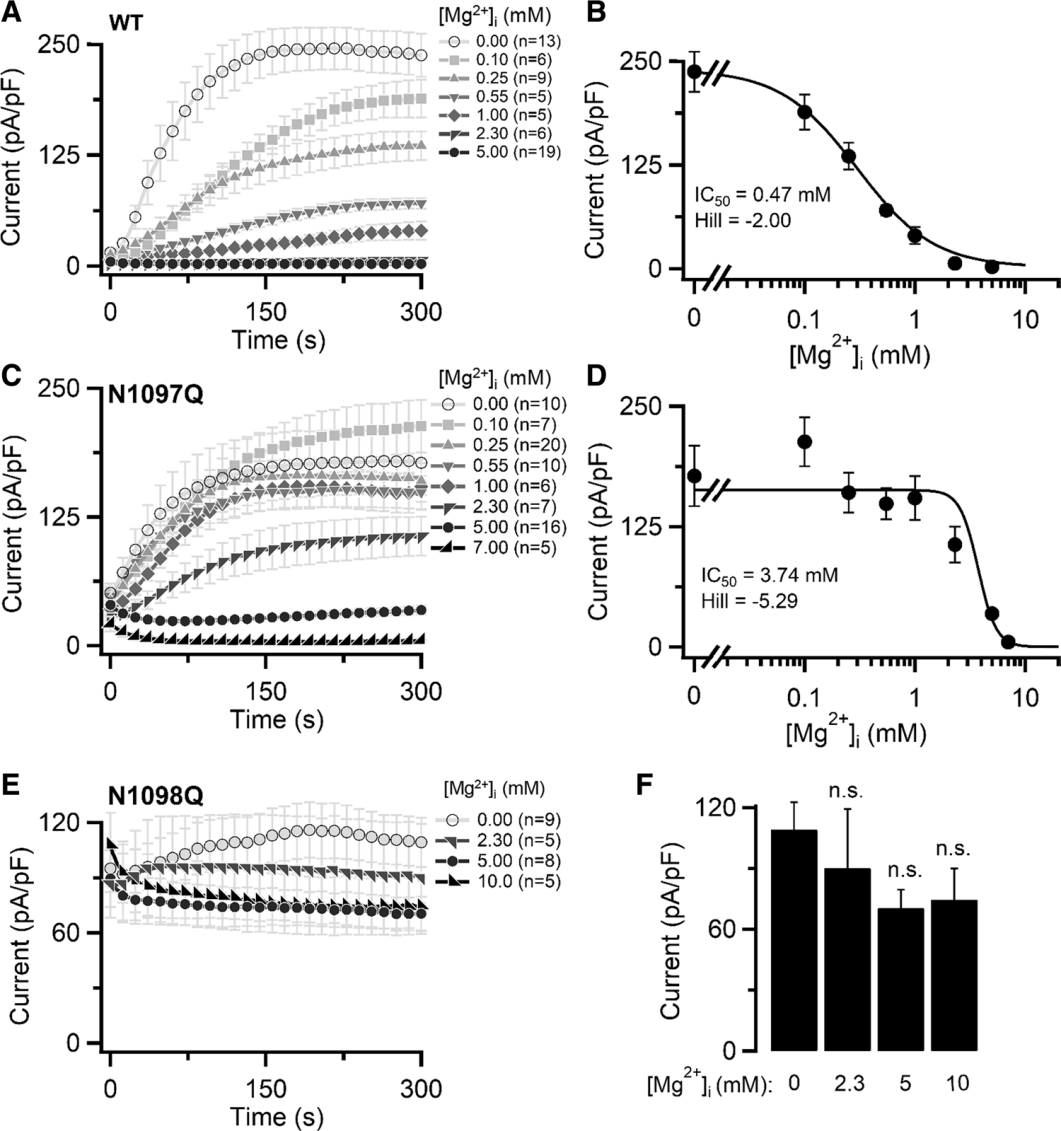
Inhibition of TRPM7 currents by cytosolic Mg^2+^. Whole-cell currents measured in HEK293T cells transfected by WT (**A, B**), N1097Q (**C, D**) and N1098Q (**E, F**) variants of TRPM7 cDNAs in pIRES2-EGFP expression vectors. **A**, **C**, **E** Current amplitudes (mean ± SEM) were measured at + 80 mV using internal solutions containing the indicated free [Mg^2+^]_i_ (Suppl. Table S1) and plotted over time. **B**, **D** Concentration-dependent suppression of currents (+ 80 mV, 300 s) shown in (**A**, **B**). The Hill equation was fitted to determine IC_50_ and the Hill factor. **F** Bar graphs of outward currents (mean ± SEM; + 80 mV) shown in (**E**) at 300 s. *n*, number of cells measured

Next, we performed patch-clamp experiments to examine the impact of N1097Q and N1098Q on the concentration-dependent suppression of the channel by free [Mg^2+^]_i_ (Fig. 2). The calculated IC_50_ value for WT currents was 0.47 mM (Fig. 2A, B). Currents in cells expressing TRPM7 carrying the N1097Q mutation were inhibited by [Mg^2+^]_i_ with an IC_50_ value of 3.74 mM (Fig. 2C, D). As [Mg^2+^]_i_ varies between 0.5 and 1.0 mM in most mammalian cells [41], these results suggest that, unlike the WT channel, the N1097Q variant remains active in the presence of physiological concentrations of Mg^2+^. Remarkably, TRPM7 containing N1098Q was highly active after break-in and remained active over time in the presence of the whole range of [Mg^2+^]_i_ examined, thus precluding a reliable calculation of an IC_50_ value (Fig. 2E, F)_._ These results indicate that the N1098Q mutation results in a constitutively active channel insensitive to physiological concentrations of intracellular Mg^2+^.

Apart from Mg^2+^, other divalent cations (Ba^2+^, Ca^2+^ and Zn^2+^) can suppress TRPM7 currents presumably through a common regulatory site [38]. Hence, we examined whether the N1097Q and N1098Q mutants interfered with the effects of free [Ba^2+^]_i_ (0.55 and 1 mM) on the TRPM7 channel variants (Figure S3). We observed that the WT TRPM7 channel was inactive in the presence of both concentrations of free [Ba^2+^]_i_ (Suppl. Fig. S3A). The N1097Q variant showed significantly reduced currents only after administration of 1 mM free [Ba^2+^]_i_ (Suppl. Fig. S3B), whereas the N1098Q channel remained unaffected under both experimental conditions (Suppl. Fig. S3C).

### Effects of N1097Q and N1098Q mutations on TRPM7 channel suppression by Mg·ATP and Mg·GTP

**Fig. 3:**
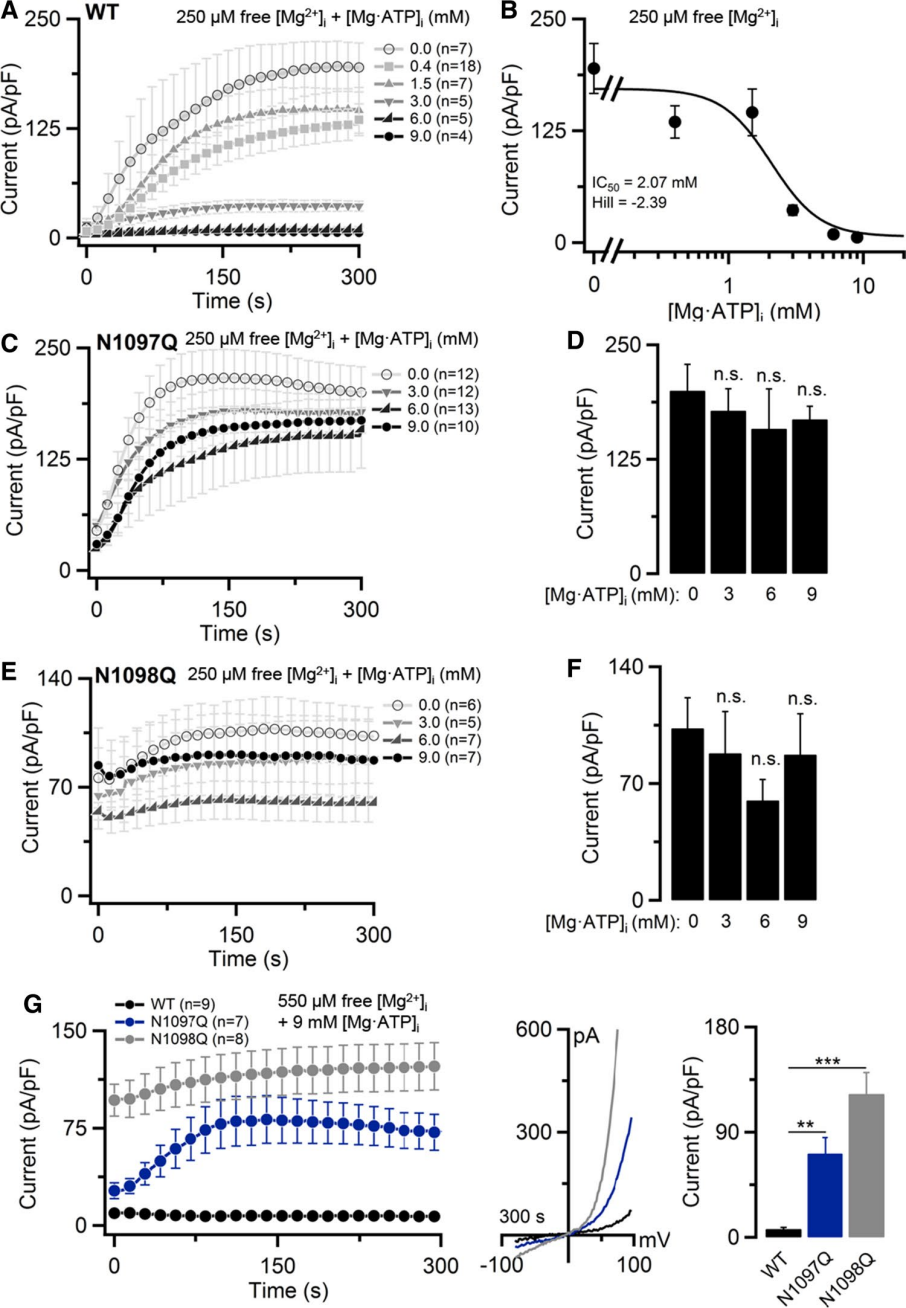
Inhibition of TRPM7 currents by cytosolic Mg·ATP. Whole-cell currents measured in HEK293T cells transfected by WT, N1097Q and N1098Q variants of TRPM7 cDNAs (in pIRES2-EGFP). **A, B** Concentration-dependent suppression of WT TRPM7 by [Mg·ATP]_i_ in the presence of 250 µM free [Mg^2+^]_i_ (Suppl. Table S3). **A** Current amplitudes of the WT channel (mean ± SEM) measured at + 80mV were plotted over time. **B** Concentration–response curve for currents shown in **A** (+ 80 mV, 300 s). The Hill equation was used to determine IC_50_ and the Hill factor. **C**–**F** Effects of [Mg·ATP]_i_ on the N1097Q (**C**, **D**) and N1098Q (**E**, **F**) variants of TRPM7. Measurements were performed as in **A**. However, bar graphs of outward currents (mean ± SEM; + 80 mV) at 300 s were used to analyse the effects of [Mg·ATP]_i_. **G** Whole-cell currents were measured in the presence of 9 mM [Mg·ATP]_i_ and 550 µM free [Mg^2+^]_i_ (Suppl. Table S4). *Left panel* current amplitudes (mean ± SEM) acquired at + 80 mV were plotted over time. *Middle panel *representative current–voltage (I–V) relationships of currents (at 300 s) illustrated in the *left panel. Right panel* bar graphs of outward currents (mean ± SEM; + 80 mV) shown in the *left panel* at 300 s. *n*, number of cells measured; ns, not significant; **P < 0.01, ***P< 0.001 (ANOVA)

Previously, extensive electrophysiological analyses revealed that free Mg^2+^ and Mg·ATP inhibit TRPM7, most likely through different ligand binding sites [12, 13]. Interestingly, the elevation of free Mg^2+^ levels increased the potency of Mg·ATP, suggesting that Mg^2+^ and Mg·ATP act synergistically on the TRPM7 channel [12, 13]. Therefore, to further verify the role of N1097Q and N1098Q, we compared the concentration-dependent inhibition of the TRPM7 channel variants by intracellular concentrations of Mg·ATP [Mg·ATP]_i_, in the presence of only 250 µM free [Mg^2+^]_i_. The physiological intracellular concentrations of ATP vary between 2 and 9 mM in most mammalian cells [39]. In a physiological saline solution, the apparent *K*_d_ of Mg·ATP is ~50 μM [40] and it was estimated that $>$90% of cytosolic ATP is present as Mg·ATP [41]. Using internal solutions covering this range of [Mg·ATP]_i_, we found that WT currents were suppressed with an IC_50_ value of 2.07 mM (Fig. 3A, B). By contrast, TRPM7 mutants N1097Q and N1098Q were characterized by a remarkably low sensitivity to [Mg·ATP]_i_ at all concentrations examined (Fig. 3C–F). Due to experimental limitations, [Mg·ATP]_i_ above 10 mM could not be reliably examined. Since other Mg·nucleotides (like Mg·GTP) were also capable of suppressing the TRPM7 channel, presumably through a mechanism shared with Mg·ATP [12, 13], we asked whether co-administration of 6 mM [Mg·GTP]_i_ and 250 μM free [Mg^2+^]_i_ will recapitulate the effects of 6 mM [Mg·ATP]_i_ co-applied with 250 μM free [Mg^2+^]_i_ (Suppl. Fig. S4). We observed that the impact of the N1097Q and N1098Q mutations on the channel’s response to both Mg·nucletides were not different in such experimental settings (Suppl. Fig. S4).

Next, we examined the effects of 9 mM [Mg·ATP]_i_ in the presence of the physiological range of [Mg^2+^]_i_ concentrations using an internal solution containing 550 µM and 1 mM free [Mg^2 +^]_i_. We found that WT currents were entirely suppressed under these conditions (Fig. 3G, Suppl. Fig. S5). In contrast, N1097Q mutant TRPM7 channel currents developed in the presence of 550 µM free [Mg^2+^]_i_ (Fig. 3G), but were undetectable after application of 1 mM free [Mg^2+^]_i_ (Suppl. Fig. S5). These results suggest that the N1097Q channel variant remained sensitive to Mg·ATP, but only in the presence of high concentrations of free Mg^2+^, in accord with the idea that Mg^2+^ and Mg·ATP independently interact with different TRPM7 channel sites [12, 13], and that the N1097Q mutation primarily affected the Mg^2+^ regulatory mechanism. The N1098Q variant was not suppressed in all experimental settings (Fig. 3E, Suppl. Fig. S5), indicating that the N1098Q mutation engendered a constitutively active channel variant.

Finally, we aimed to assess the activity of TRPM7 variants without manipulations of the cytosolic contents of Mg^2+^ and Mg·ATP using perforated patch recordings (Suppl. Fig. S6). Consistent with earlier studies [21], WT currents did not develop in this experimental setting, presumably because resting concentrations of Mg^2+^ and Mg·ATP are sufficient to inhibit TRPM7. On the contrary, channel activity of the N1097Q or N1098Q variants was well detectable (Suppl. Fig. S6), supporting the notion that the N1097Q or N1098Q mutations diminish the inhibitory effects of both Mg^2+^ and Mg·ATP in resting HEK293T cells.

### Effects of N1097Q and N1098Q on the sensitivity of TRPM7 to pharmacological agents and PIP_2_ depletion

**Fig. 4:**
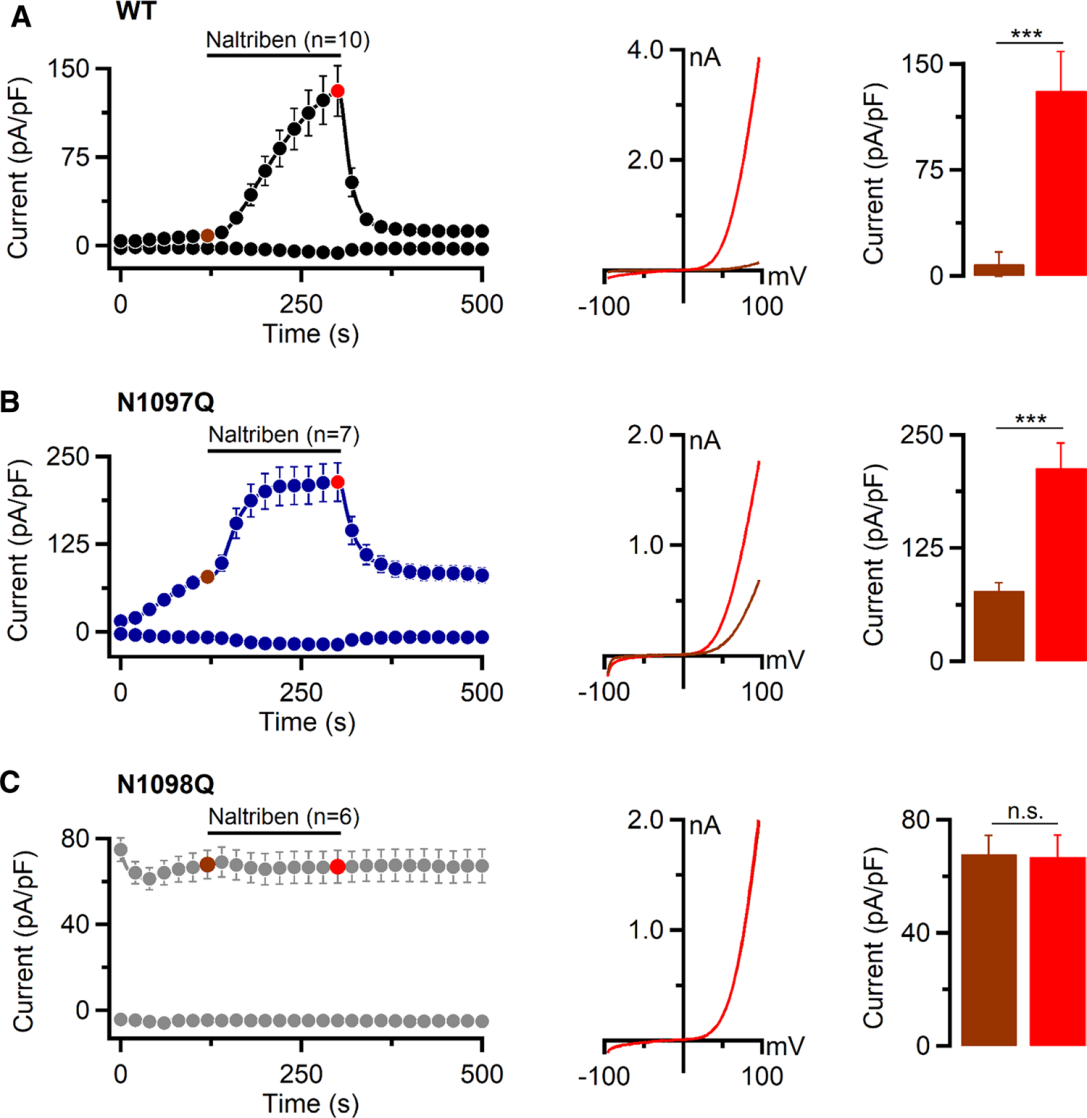
Activation of TRPM7 currents by naltriben. Whole-cell currents were measured in HEK293T cells transfected by WT (**A**), N1097Q (**B**) and N1098Q (**C**) variants of TRPM7 cDNAs (in pIRES2-EGFP). *Left panels* current amplitudes (mean ± SEM) were measured at − 80 and + 80 mV and plotted over time. Currents were measured using an intracellular solution containing 9 mM [Mg·ATP]_i_ and 550 µM [Mg^2+^]_i_ and the standard external solution with or without 100 µM naltriben as indicated by the black bars. *Middle panels* representative I–V relationships obtained from individual ramps before (brown) and after (red) naltriben application as indicated in the *left panels* by coloured data points. *Right panels* bar graphs of outward currents (+ 80 mV, mean ± SEM) obtained before (brown) and after (red) naltriben application as indicated in the *left panels* by coloured data points. *n*, number of cells measured; ns, not significant; ***P< 0.001 (two-tailed *t* test)

To determine whether the N1097Q or N1098Q amino acid exchanges altered the sensitivity of TRPM7 exclusively to [Mg^2+^]_i_ and [Mg·ATP]_i_ or caused more general changes of regulatory characteristics of the channel, we studied the effects of small synthetic molecules acting as activators or inhibitors of the TRPM7 channel. First, we assessed the action of naltriben, a potent agonist of the TRPM7 channel [42]. In these experiments, we used intracellular solutions containing 9 mM [Mg·ATP]_i_ and 550 µM free [Mg^2+^]_i_. The external application of naltriben led to a fast stimulation of WT and N1097Q currents (Fig. 4A, B). The N1098Q variant did not respond to naltriben (Fig. 4C).

**Fig. 5:**
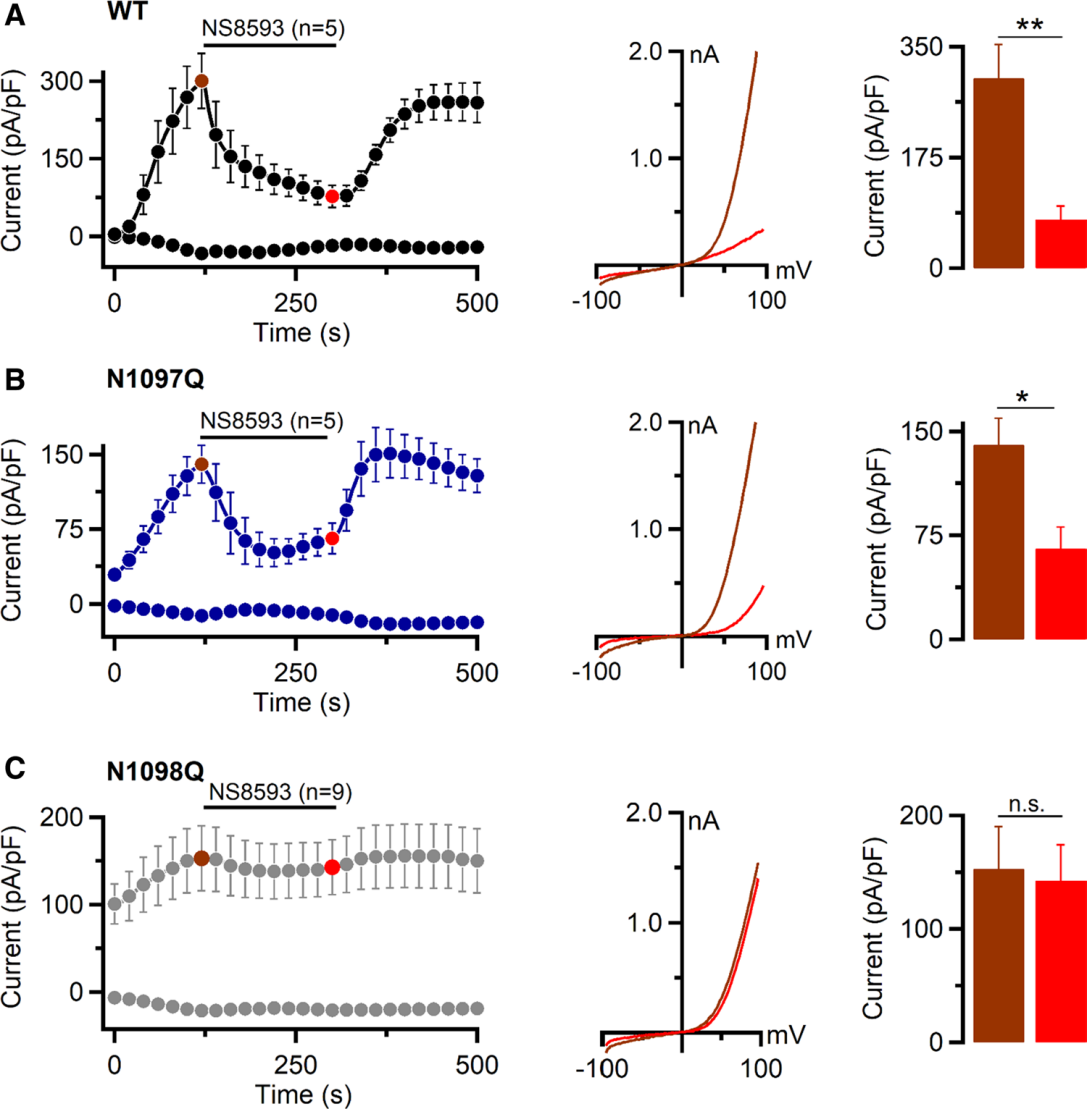
Effects of NS8593 on TRPM7 currents. Whole-cell currents were measured in HEK293T cells transfected by WT (**A**), N1097Q (**B**), and N1098Q (**C**) variants of TRPM7 cDNAs (in pIRES2-EGFP) expressed in HEK293T cells. *Left panels* current amplitudes (mean ± SEM) were acquired at − 80 and + 80 mV and plotted over time. Currents were induced using the standard [Mg^2+^]_i_-free intracellular solution and the standard external solution. When currents were activated, the cells were exposed to the standard external solution with 10 µM NS8593 as indicated by the black bars. *Middle panels* representative I–V relationships obtained from individual ramps before (brown) and after (red) NS8593 application as indicated in the *left panels* by coloured data points. *Right panels* bar graphs of outward currents (+ 80 mV, mean ± SEM) obtained before (brown) and after (red) NS8593 application as indicated in the *left panels* by coloured data points. *n*, number of cells measured; ns, not significant; *P< 0.05; **P< 0.01 (two-tailed *t* test)

Next, we examined the effect of NS8593, a potent TRPM7 inhibitor [43]. In these experiments, we induced currents using the standard [Mg^2+^]_i_-free intracellular pipette solution and externally applied NS8593 when currents were fully developed (Fig. 5). We noted that NS8593 caused a rapid inhibition of WT and N1097Q currents (Fig. 5A, B). In analogy to the effects of naltriben (Fig. 4A), the N1098Q mutation abolished the channel's sensitivity to NS8593 (Fig. 5C), indicating that the N1098Q mutant is functionally different from the N1097Q variant with regard to its sensitivity to natural or synthetic ligands.

**Fig. 6:**
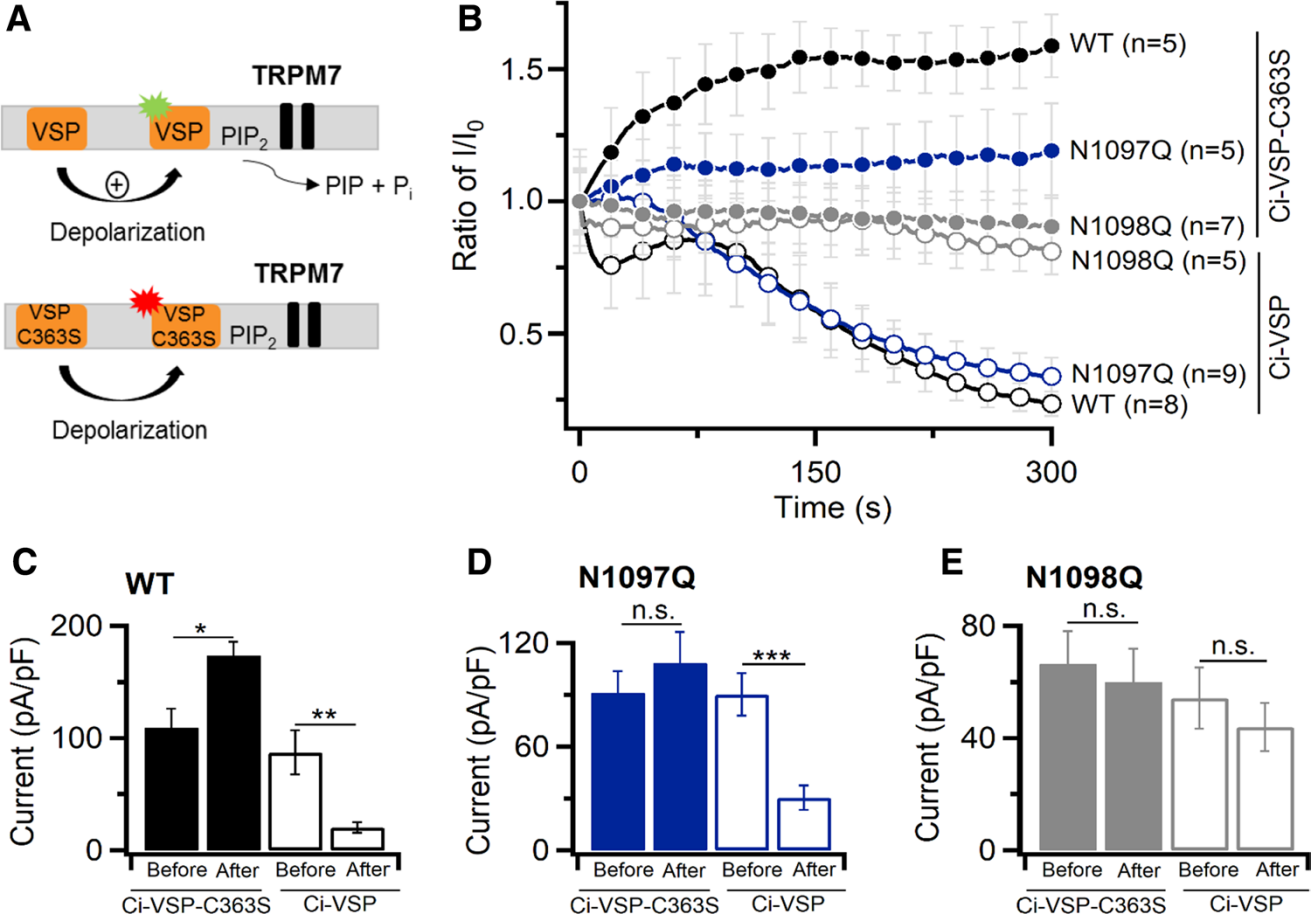
Assessment of TRPM7 currents after PIP_2_ depletion. **A** A diagram showing how wild-type voltage-sensitive phosphatase from *Ciona intestinalis* (Ci-VSP) reduces PIP_2_ levels in the plasma membranes. A catalytically silent mutant of Ci-VSP (Ci-VSP-C363S) is unable to affect PIP_2_ contents. **B** Time-dependent changes of normalized whole-cell outward currents in HEK293T cells transfected with WT, N1097Q and N1098Q variants of TRPM7 cDNA and Ci-VSP or Ci-VSP-C363S. Cells were held at − 60 mV for 2 min to allow induction of TRPM7 without activation of Ci-VSP. Then the voltage ramp ranging from − 100 to + 100 mV was applied to activate Ci-VSP and record TRPM7 currents. Current amplitudes (+ 80 mV) were normalized to the initial currents (mean ± SEM) and plotted over time. Data points are shown for every 10's ramp. *n*, number of cells measured. **C**–**E** Bar graphs of outward currents (+ 80 mV, mean ± SEM) shown in **B** immediately and after 300 s of application of voltage ramps from − 100 to + 100 mV. ns, not significant; *P< 0.05; **P< 0.01; ***P< 0.001 (two-tailed *t* test)

It is well documented that depletion of plasma membrane PIP_2_ results in the inactivation of the TRPM7 channel [20–22]. Therefore, we investigated the response of the TRPM7 variants to such a treatment using a voltage-sensitive phosphatase from *Ciona intestinalis* (Ci-VSP) [44]. Ci-VSP reduces PIP_2_ levels in the plasma membranes at positive membrane potentials (Fig. 6A). A catalytically silent mutant of Ci-VSP (Ci-VSP-C363S) cannot hydrolyse PIP_2_ and was used as a control (Fig. 6A). Cells co-expressing Ci-VSP or Ci-VSP-C363S together with TRPM7 variants were held at − 60 mV to allow induction of TRPM7 currents without activation of Ci-VSP. Then the regular voltage ramp ranging from − 100 to + 100 mV was applied to activate Ci-VSP and record TRPM7 currents (Fig. 6B). We found that Ci-VSP, but not Ci-VSP-C363S, suppressed WT and N1097Q currents similarly (Fig. 6C, D). In contrast, N1098Q currents were not affected by Ci-VSP and Ci-VSP-C363S (Fig. 6E). Hence, the N1097Q channel resembles the WT channel in the sensitivity to naltriben, NS8593 and PIP_2_ depletion by Ci-VSP, whereas the N1098Q variant represents a constitutively active channel insensitive to these agents.

### Assessment of TRPM7 channel variants in a divalent cation-free (DVF) extracellular solution

A well-known characteristic feature of TRPM7 is the permeation block of the channel pore by extracellular divalent cations [12], causing the characteristic shape of the I–V relationship of TRPM7 currents in the presence of divalent cations in external solutions (Fig. 1C). However, exposure of TRPM7-expressing cells to a DVF solution abolish such a permeation block and entails large monovalent cation currents with a distinguishing semi-linearized I–V relationship [12]. We examined whether N1097Q or N1098Q would affect this channel feature. As illustrated in Suppl. Fig. S7, application of DVF solution to the WT channel caused rapid increases of outward and inward currents accompanied with expected alterations in the I–V relationships. After the removal of the DVF solution, this characteristic I–V relationship of TRPM7 was reversed. We also noted that the N1097Q or N1098Q variants recapitulated the response of WT TRPM7 (Suppl. Fig. S7), indicating that N1097Q or N1098Q did not impinge on the function of the ion selectivity filter of TRPM7.

### Impact of N1097Q on TRPM7 characteristics in excised outside-out patches

For a more thorough examination of the hypothesis that the N1097Q mutation affected the sensitivity of TRPM7 to intracellular Mg^2+^, we thought to analyse TRPM7 on the single-channel level. However, our extensive attempts to measure TRPM7 currents in inside-out patches were unsuccessful. Therefore, we used previously established experimental settings to analyse TRPM7 activity in excised outside-out patches [45]. To this end, outside-out patches were excised from transfected HEK293 cells and voltage clamped to − 60 mV. TRPM7 activity was evoked by exchanging the standard bath solution with a divalent cations-free (no-DIV) solution. This manoeuvre elicited only multichannel responses, irrespective of whether patches were from WT- or N1097Q-expressing cells, precluding an analysis of single-channel kinetics in these experimental settings (Suppl. Figure 8A, B). Nevertheless, differences between the WT and N1097Q channels were well evident. Single-channel amplitudes and the sojourns in the open state were about twice as large in the WT channels (Suppl. Fig. S8A, B). Estimated form a subset of experiments in which current–voltage relationships could be obtained, the calculated single-channel slope conductances (*γ*_s_) were 41.4 ± 2.5 pS and 23.8 ± 2.4 pS for the WT and N1097Q, respectively [*n* = 7; see Suppl. Fig. S8C for examples; Suppl. Table S5 provides auxiliary chord conductance values (*γ*_c_) from a larger sample]. The corresponding surrogate mean open times (*T*_OS_), an admittedly rough estimate for the duration of the open state, were 4.7 ± 0.6 ms and 1.5 ± 0.3 ms for the WT and N1097Q channel variants, respectively. Despite shortening of *T*_OS_, N1097Q did not affect the open probability (*NP*_O_) (Suppl. Fig. S8C and Table S5), suggesting that the N1097Q mutation facilitated gating motions in the TRPM7 protein. Interestingly, besides a 40 pS main level, at least one additional subconductance state for TRPM7 has been described [46]. However, the all-points histograms derived from our data (Fig. 5A, C) showed evenly spaced peaks and lack of overt humps and, therefore, do not point to a significant contribution of such a subconductance state to the overall conductance under our experimental conditions.

**Fig. 7:**
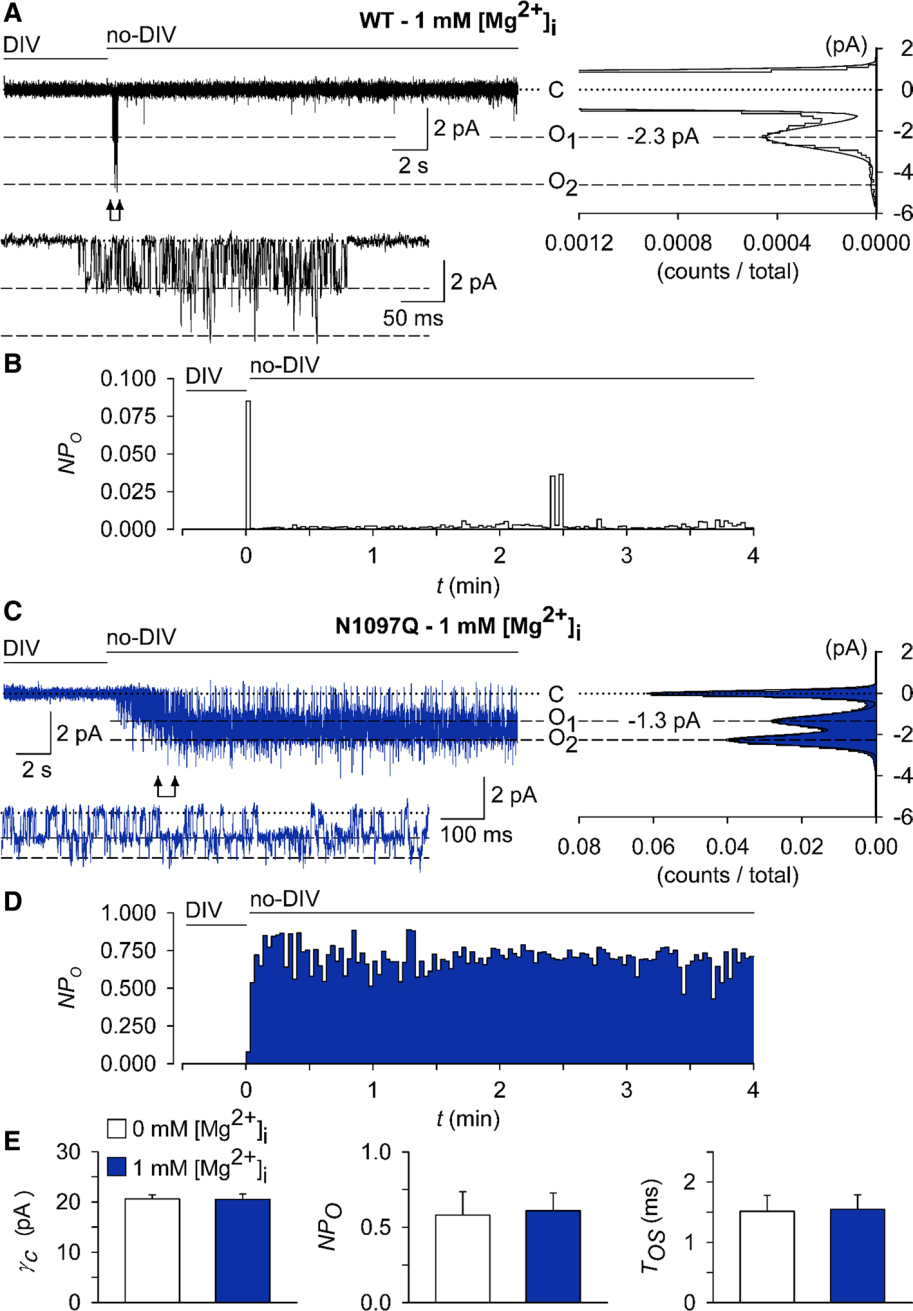
Impact of intracellular Mg^2+^ on single-channel currents from TRPM7 variants. Currents were recorded at a holding potential of − 60 mV in outside-out membrane patches excised from HEK293 cells expressing WT (**A**, **B**) and N1097Q (**C**–**E**) variants of TRPM7. The WT (**A**) or N1097Q (**C**) channels were unblocked by removing the extracellular divalent cations (DIV bath) using a no-DIV solution, as indicated above the current traces. The intracellular solution was no-DIV augmented by 1 mM Mg^2+^. Insets: currents on an expanded time scale from the segments indicated by arrows. The graphs on the right show all-point histograms from the two 30 s current traces. The dotted line indicates the closed level (**C**). The broken lines indicate the current level for one channel (*O1*) or two channels (*O2*) being open. Single-channel amplitudes (i) taken from O1 were − 2.3 pA and − 1.3 pA for WT channels (**A**) and N1097Q (**C**), respectively. **B**, **D** The open probabilities *(NP*_*O*_) assessed for bins of 2 s over the whole 4.5 min duration of the experiments shown in (**A**, **C**). Note the different ordinate scaling in **B** and **D**. **E** Statistical evaluation of outside-out recordings with the N1097Q channel. Note that intracellular Mg^2+^ did not affect single-channel chord conductance (*γ*_c_), NP_O_ and open time (*T*_OS_; *n* = 7 each)

To assess the effects of intracellular Mg^2+^ on channel characteristics, we used a pipette solution containing 1 mM free Mg^2+^. All outside-out patches from cells overexpressing the WT channel exposed to no-DIV solution showed only an initial burst of channel openings followed by lasting quiescence (Fig. 7A, B). Single-channel current amplitudes assessed during the initial outbreaks of activity (− 2.3 ± 0.18 pA, Fig. 7A) were similar to those obtained in the absence of intracellular Mg^2+^ (− 2.2 ± 0.21 pA; Suppl. Fig. S8, Table S5). N1097Q behaved differently from the WT channel. Exposure to no-DIV solution induced sustained channel activity of the N1097Q variant, with the *NP*_O_ being stable over the whole time of current recordings (Fig. 7C, D). All measured channel characteristics, including chord conductance, *NP*_O_, and *T*_OS_ were not affected by adding 1 mM intracellular Mg^2+^ (Fig. 7E, Table S5). Finally, using the same experimental settings, we analysed the effects of 2.9 mM [Mg·ATP]_i_ and 250 μM free [Mg^2+^]_i_ and observed that the WT channel exhibited only an initial burst of channel openings, whereas the N1097Q variant was active and displayed functional characteristics similar to those obtained in the presence of 1 mM free Mg^2+^ (Suppl. Fig. S9). These results corroborate with our analysis of whole-cell currents reinforcing the idea that the N1097Q substitution abrogated the inhibition of the TRPM7 channel by physiological intracellular Mg^2+^ concentrations.

### Modelling and molecular dynamics (MD) simulations of the wild-type and mutant variants of the TRPM7 channel in closed and open conformations

**Fig. 8:**
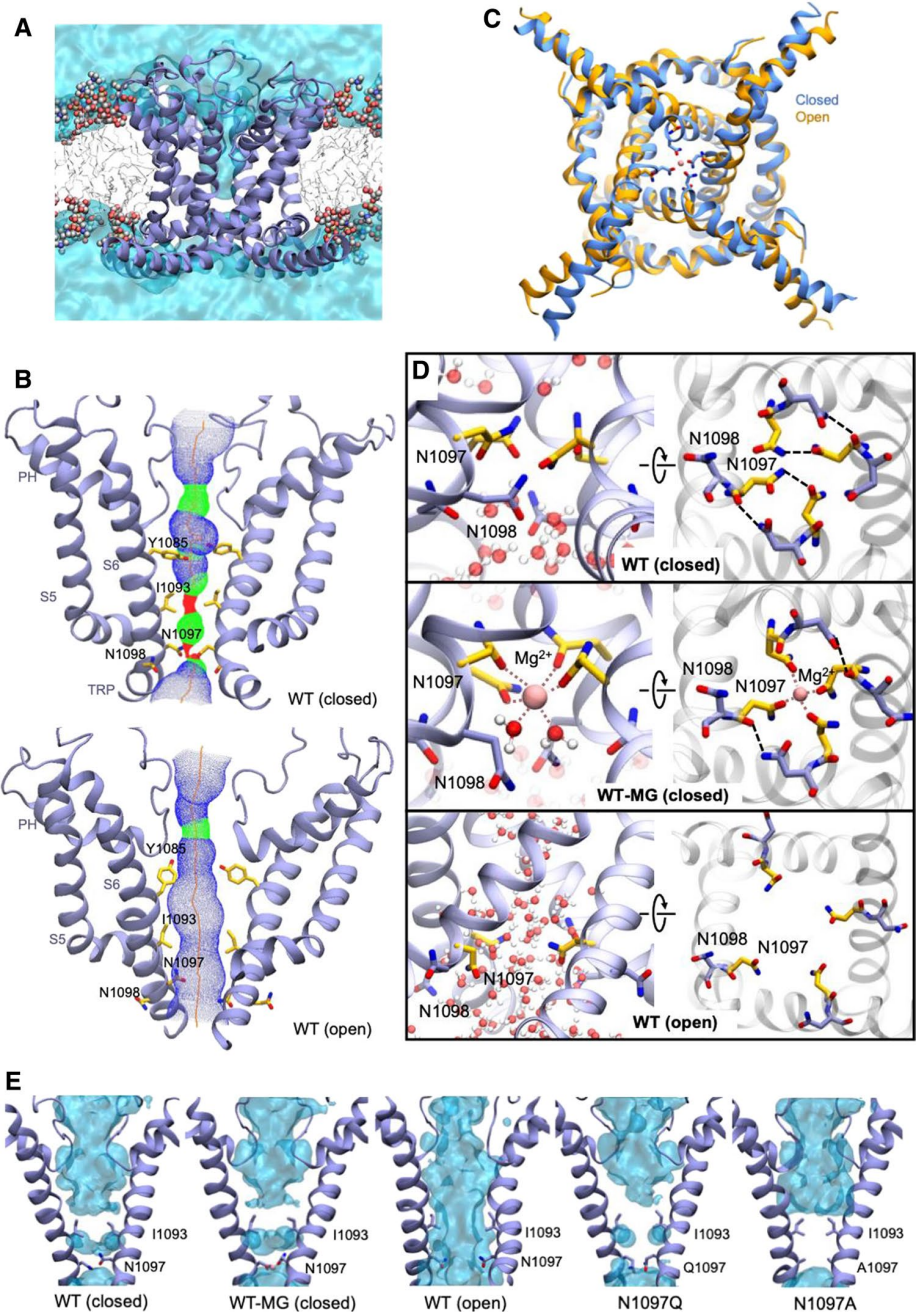
Models of the channel pore-forming segment of mouse TRPM7 in lipid and water environments in closed and open conformations. **A** A representative structure of the mouse WT TRPM7 simulated channel unit (residues 982 to 1123) in the lipid bilayer and water. **B** A side view of a channel structure in the closed (top panel) and open (bottom panel) conformations. The channel pore-lining residues are shown. **C** Overlap of the closed structure an open structure shown (bottom view). **D** Structure of the channel gate in the closed channel with or without Mg^2+^ and open channel. Persistent water molecules are shown in red. Dashed lines indicate hydrogen bonds formed between N1097 and N1098 residues and Mg^2+^. Note that the hydrogen bonds are transient, and the specific interactions shown are not present in all frames of the trajectory. **E** Channel’s hydration in the simulations is shown for the WT, N1097A and N1097Q variants. Water is shown as a semi-transparent light blue continuum

To elucidate a mechanism by which N1097 and N1098 contribute to TRPM7 channel regulation, we opted for protein modelling and MD simulations of the TRPM7 ion channel domain. The TRPM7 structure in Mg^2+^-free conditions (PDB 5ZX5) showed the highest resolution of the S5–S6 segment in TRPM7, and, therefore, it was selected for analysis. To perform MD simulations, we constructed a 3D model of the closed channel pore-forming segment of TRPM7 (S4–S5 linker, S5–S6 helices and TRP domain) embedded in a lipid membrane and surrounded by water and ions (Fig. 8A). To develop a TRPM7 open channel structure, we used the homology modelling method. Initially, we attempted to use the open structure of the zebrafish TRPM2 channel as a template [32]. However, the open TRPM2 channel displayed significant conformational changes in the S5 helix driven by the interaction of charged residues in the S4–S5 linker and the TRP box, which are not conserved in TRPM7. Therefore, our analysis relied on the open structure of the human TRPV6 channel (PDB: 6BO8 [47]) (Suppl. Fig. S10).

The homology-modelled open channel structure then was used in targeted MD simulations to open the channel in lipid and water (Fig. 8). We observed that in the closed state of the channel, the side chains of I1093 formed a hydrophobic seal excluding water from the channel (Fig. 8E), whereas polar side chains of N1097 build the narrowest segment of the cation permeation path in the pore (Fig. 8B). We found that transient inter-subunit hydrogen bonds were formed between N1097 residues of different subunits and between N1097 and N1098 residues. The most prominent hydrogen bonds were formed between the side chain amino and carbonyl groups of N1097 residues of adjacent subunits and between the side chain amino group of N1098 and the backbone carbonyl group of N1097 of adjacent subunits (Fig. 8D). On average, 3.6 inter-subunit hydrogen bonds involving N1097 and N1098 were present in the tetramer during the simulation (Suppl. Table S6). Our analysis suggests that these hydrogen bonds serve to stabilize the closed state of the channel gate (Fig. 8B, D).

MD simulation of TRPM7 in the open channel state revealed several remarkable structural rearrangements compared to the closed pore state (Fig. 8B, D). Specifically, we observed a loss of the hydrophobic seal formed by I1093 and significantly increased distance between the side chains of N1097. Inter-subunit hydrogen bonds formed by N1097 and N1098 were nearly absent with an average of 0.36 (Suppl. Table S6). Next, we performed MD simulations with Mg^2+^ placed in the pocket formed by the four N1097 in the closed channel (Fig. 8D). We found that Mg^2+^ steadily interacted with the side chain carbonyl groups of N1097 and two water molecules (Fig. 8C, D), stabilizing the closed state of TRPM7.

Finally, we performed MD simulations of the N1097A, N1097Q, N1098Q mutants. The N1097A channel formed a tightly closed gate due to the hydrophobicity of A1097 downstream to the hydrophobic seal formed by I1093 (Fig. 8E), thus explaining the silencing of the channel variant in patch-clamp experiments (Fig. 1). In the absence of Mg^2+^, the N1097Q variant behaved similarly to the WT channel (Fig. 8E). However, MD simulations in the presence of Mg^2+^ revealed that Mg^2+^ lost coordination with three out of the four side chains of Q1097 (Suppl. Figure 11A), thus explaining the shift in concentration–response Mg^2+^ inhibition of the N1097Q channel (Fig. 2). The N1098Q presented a more complicated picture. In the absence of Mg^2+^, the N1098Q mutation reduced the average number of inter-subunit hydrogen bonds formed by N1097 and Q1098 to only 0.6 as opposed to 3.6 in the WT structure (Suppl. Table S6, Suppl. Figure 11B). These results suggest that the closed state of the N1098Q channel is significantly less stable than the WT channel. In the presence of Mg^2+^, the N1098Q channel retained coordination with Mg^2+^, but the glutamine residue at position 1098 interacted weaker with N1097 than the WT channel (Suppl. Table S6). Hence, consistent with electrophysiological data, N1097 and N1098 have distinct structural roles in the lower channel gate of TRPM7.

## Discussion

There is growing evidence to show that TRPM7 is a central gatekeeper of the cellular uptake of essential divalent cations and that cytosolic Mg^2+^ acts as the principal regulator of this fundamental process. In the present paper, we used a combination of site-directed mutagenesis, electrophysiological techniques and MD simulations to show that side chains of N1097 in mouse TRPM7 form an inter-subunit Mg^2+^-regulatory site, determining the responses of the channel to changes of cytosolic Mg^2+^ levels. Hence, our study offers a molecular explanation of the key regulatory characteristic of the TRPM7 channel. The primary role of the lower channel gate in sensing the intracellular milieu has not been described yet in TRPM channels. Whether such a mechanism epitomizes a general principle among other TRP channels remains to be answered in the future.

The current view is that intracellular Mg^2+^ and Mg·ATP represent physiologically relevant negative regulators of the channel. Because TRPM7 contains a kinase unit, initial studies attempted to address the regulatory role of this domain [13–15, 21]. However, a kinase-dead point mutation (K1646R in mouse TRPM7) or channel variants lacking the C-terminal segments of the channel, including the kinase moiety, resulted in channels with only modestly changed sensitivity to Mg^2+^ [13–15, 21]. In contrast, the effects of Mg·ATP were dependent on the kinase moiety and upstream coiled-coil segments of TRPM7 in a species-specific fashion [13–15, 21]. These findings can be interpreted to mean that Mg^2+^ and Mg·ATP operate through different regulatory sites in TRPM7 and that Mg^2+^ blocks TRPM7 independently from the kinase domain.

In the present study, we uncovered the structural basis of the inhibitory action of free Mg^2+^ on the TRPM7 channel. Our hypothesis-driven assessment of TRPM7 variants with point mutations spanning different segments of TRPM7 suggests that the lower channel gate determines the Mg^2+^ sensitivity of TRPM7. Thus, a slight modification of the side chain of N1097 (N1097Q mutation) was sufficient to shift the IC_50_ value beyond physiological levels of free Mg^2+^. Consequently, the N1097Q mutant was active in perforated patch-clamp recordings when the patch pipette solutions did not manipulate the intracellular Mg^2+^ levels. Interestingly, the N1097Q channel showed reduced sensitivity to free Ba^2+^, implying that other divalent cations can potentially occupy the proposed Mg^2+^ binding site, thus, providing a mechanistic explanation for the inhibition of TRPM7 currents by Ba^2+^, Ca^2+^ and Zn^2+^ when applied in the mM range [38]. Significantly, N1097Q did not affect the current amplitudes of the channel in the absence of intracellular or extracellular Mg^2+^ and displayed unchanged I–V characteristics in all experimental settings used. The N1097Q mutant retained the sensitivity to pharmacological agents acting as negative and positive gating modulators of TRPM7, such as NS8593 and naltriben. Moreover, assessing the biophysical properties of the N1097Q mutant on the single-channel level corroborated our conclusion derived from whole-cell data, in that the mutant channel remains active in the presence of physiological concentrations of internal Mg^2+^. Collectively, these results indicate that the N1097Q mutation selectively affects the inhibitory action of intracellular Mg^2+^ rather than perturbing TRPM7 function unspecifically.

Although the present study primarily aimed to reveal regulatory mechanisms of Mg^2+^, our experiments also provide new insight into the action of PIP_2_ and Mg·ATP on TRPM7. Thus, the N1097Q variant responded to PIP_2_ depletion similarly to the WT channel_._ The latter finding is not surprising because the side chain of N1097 is exposed to the channel pore lumen and, consequently, incapable of interacting directly with membrane PIP_2_. However, we observed that the N1097Q channel was sensitive to unphysiologically high Mg^2+^ concentrations suggesting that an additional action of Mg^2+^ was retained, for instance, the predicted electrostatic Mg^2+^ shielding of negatively charged PIP_2_ [20–22]. Also, we observed that in the presence of relatively low Mg^2+^ levels (250 and 550 μM), the N1097Q variant exhibited a significantly reduced sensitivity to Mg·ATP (and Mg·GTP). However, the inhibitory effect of Mg·ATP on N1097Q currents was retained in the presence of 1 mM free Mg^2+^. These results are consistent with a previous study [13], demonstrating that free Mg^2+^ and Mg·ATP can interact with TRPM7 causing the synergistic inhibition of TRPM7 currents. Accordingly, we suggest that the N1097Q mutation primarily affects the channel’s response to free Mg^2+^, while its sensitivity to Mg·ATP is still preserved.

MD simulations allowed us to interrogate the structural role of N1097 in opening of the TRPM7 channel. Our data suggest that the four side chains of N1097 in a TRPM7 tetramer form an inter-subunit cation-binding site and that the presence of Mg^2+^ in this pocket stabilizes the closed channel state. Accordingly, the lack of Mg^2+^ facilitates channel opening. In line with electrophysiological experiments, the N1097Q variant eradicates the coordination of Mg^2+^ in the lower channel gate due to the difference of one methylene group in the length of side chains of asparagine and glutamine, thus destabilizing the closed channel state in the presence of Mg^2+^. Unlike N1097Q, a similar modification of an adjacent asparagine, N1098Q, resulted in a gain-of-function mutation completely offsetting the effects of Mg^2+^, Ba^2+^, Mg·ATP, Mg·GTP, PIP_2_ and pharmacological agents. Such constitutive activity of the N1098Q channel resembles a previously isolated TRPM7 variant containing the point mutation S1107E in the TRP domain [42]. The S1107E channel was insensitive to physiological levels of free Mg^2+^, PIP_2_ and naltriben [42].

The structural role of N1098 is distinguishable from that of N1097. MD simulations suggest that N1098 forms inter-subunit hydrogen bonds stabilizing the closed state of the WT channel. Consequently, the N1098Q mutation most likely destabilizes such hydrogen bonds resulting in a constitutively active channel variant. The striking functional impact of N1098Q and the closely located S1107E further reinforce the notion that this segment of TRPM7 plays a crucial role in opening of the channel.

Previously, our structure–functional analysis of mouse TRPM7 identified the crucial role of E1047 in the cation selectivity filter of the channel [48]. Together with other researchers [23, 48, 49], we showed that the E1047Q variant of TRPM7 was essentially impermeable to divalent cations, supporting the idea that the side chain of E1047 forms an inter-subunit site that directly interacts with divalent cations entering the channel pore. To this end, the structural impact of E1047 in the cation selectivity filter resembles the role of N1097 in the lower channel gate of TRPM7, implying the channel function of TRPM7 primarily operates using two inter-subunit cation-binding sites interacting with extracellular and cytosolic divalent cations. Since N1097 and N1098 of mouse TRPM7 are conserved within the TRPM1/3/6/7 group of mammalian proteins (Fig. 1G), such a structure–function paradigm may be relevant for this subgroup of TRPM channels as a general principle.

## Material and methods

### Molecular biology and cell culture

Mouse TRPM7 (in pIRES2-EGFP vector) and TRPM7-YFP (in pcDNA3.1/V5-His TA-TOPO vector) were reported previously [36, 37]. Ci-VSP or Ci-VSP-C363S cDNAs (in pIRES2-EGFP) were provided by Joris Vriens, KU Leuven [50].

Point mutations in TRPM7 were introduced using the QuikChange system (Thermo Fisher Scientific) according to the manufacturer’s protocol and verified by sequencing (Eurofins, Germany). Initially, we attempted to introduce the mutations outlined in Fig. 1A in the mouse TRPM7 cDNA in the bicistronic pIRES2-EGFP vector [36, 37]. However, we found that side-directed mutagenesis of this expression construct is highly inefficient and prone to errors likely due to cis-acting IRES sequence. Nevertheless, we could successfully conduct mutagenesis using the mouse TRPM7 with C-terminal YFP tag in pcDNA3.1 vector [36, 37] and, consequently, the primary functional assessment of TRPM7 variants (Fig. 1) was performed using the latter expression construct.

HEK293T cells were grown at 37 °C and 5% CO_2_ in Dulbecco’s modified Eagle’s medium (DMEM, Sigma-Aldrich) supplemented with 10% foetal bovine serum (FBS, Thermo Fisher Scientific), 100 U/ml penicillin and 100 µg/ml streptomycin (P/S, Sigma-Aldrich). Cells ~60% confluence, 3 cm dish) were transiently transfected by 2 µg TRPM7 cDNAs using Lipofectamine 2000 reagent (Thermo Fisher Scientific). In some experiments, 2 µg TRPM7 cDNAs (pIRES2-EGFP) were co-transfected with 1 µg Ci-VSP WT or Ci-VSP-C363S cDNAs (pIRES2-EGFP) as indicated in the corresponding figure legend.

### Immunofluorescent staining

HEK293T cells cultured on glass-bottom cell culture dishes (World Precision Instruments) were transiently transfected by 2 µg/dish WT or mutant variants of TRPM7 cDNA (in pIRES2-EGFP) and examined 18–24 h after transfection. Cells were washed twice with PBS, fixed with ice-cold methanol for 20 min at − 20 °C, and blocked for 1 h with 5% (v/v) BSA in PBS at room temperature. The mouse monoclonal anti-TRPM7 antibody (clone 2C7; 0.84 μg/ml; [51]) was applied. The secondary antibody (0.5 μg/ml) was goat anti-mouse IgG conjugated to Alexa Fluor 488 (Molecular Probes). Each incubation was performed in PBS containing 5% (v/v) normal goat serum for 1 h at room temperature, followed by triple washing with PBS. After the final washing, glass coverslips were placed on glass-bottom cell culture dishes using a mounting medium (DakoCytomation). Differential interference contrast (DIC) and Airyscan images of Alexa Fluor 488 were obtained with the confocal laser-scanning microscope LSM 880 AxioObserver (Carl Zeiss). We used a C-Apochromat 63x/1.2 W objective, 488 nm excitation wavelength and 493–630 nm filters, multi-line argon laser 458/488/514 nm and an Airyscan detector. The acquired images were analysed using the ZEN 3.0 SR software (Carl Zeiss).

### Electrophysiological techniques

Patch-clamp experiments with HEK293T cells were performed 18–22 h after transfection as reported previously [37, 52] with a few modifications. Whole-cell currents were measured using an EPC10 patch-clamp amplifier and PatchMaster software (Harvard Bioscience). Voltages were corrected for a liquid junction potential of 10 mV. Currents were elicited by a ramp protocol from − 100 mV to + 100 mV over 50 ms acquired at 0.5 Hz and a holding potential of 0 mV. Inward and outward current amplitudes were extracted at − 80 mV and + 80 mV and were normalized to cell size as pA/pF. Capacitance was measured using the automated capacitance cancellation function of EPC10. Patch pipettes were made of borosilicate glass (Science Products) and had resistance 2–3.5 MΩ.

Unless stated otherwise, a standard extracellular solution contained (in mM): 140 NaCl, 2.8 KCl, 1 CaCl_2_, 2 MgCl_2_, 10 HEPES–NaOH and 11 glucose (all from Roth Industries), pH 7.2. Effects of NS8593 (Tocris) and naltriben (Tocris) were examined by adding the compounds to the standard extracellular solution. A divalent cation-free (DVF) extracellular solution contained (in mM) 140 NaCl, 2.8 KCl, 11 glucose, 10 Na-EDTA and 10 HEPES–NaOH, pH 7.2. For the experiment with TRPM7-YFP (Fig. 1), we used an extracellular solution contained (in mM): 140 NaCl, 2.8 KCl, 1 CaCl_2_, 10 HEPES–NaOH, and 11 glucose (all from Roth Industries), pH 7.2. The standard Mg^2+^-free intracellular ([Mg^2+^]_i_) pipette solution containing (in mM): 120 Cs·glutamate, 8 NaCl, 10 Cs-EGTA, 5 Cs-EDTA, 10 HEPES–CsOH, pH 7.2.

To obtain [Mg^2+^]_i_ and [Mg·ATP]_i_ concentration–response data, the intracellular pipette solutions were prepared as outlined in Suppl. Tables S1–S4. Concentrations of [Mg·ATP]_i_ and free [Mg^2+^]_i_ were calculated using the Maxchelator software (maxchelator.stanford.edu).

The concentration–response data were fitted (Prism 8.4.0) with the following equation:

$$E(c) = E_{{{\text{min}}}} + (E_{{{\text{max}}}} - E_{\min } ) \times (1/(1\; + ({\text{IC}}_{50} /c)^{h} )),$$

with *E* being the effect/current at a given concentration *c* of inhibitor; *E*_min_, the minimal effect/current; *E*_*m*ax_, the maximal effect; IC_50_, the half-maximal concentration; *h*, the Hill factor.

For perforated patch recordings, 320 µM amphotericin B (Sigma-Aldrich) was added to the internal solution containing (in mM): 120 monopotassium glutamate (Sigma-Aldrich), 8 NaCl, 1 MgCl_2_, 10 HEPES–KOH, pH 7.2. Cells were held at − 60 mV for 2 min to ensure activation of TRPM7 currents, followed by the standard voltage ramp protocol.

Outside-out patch-clamp recordings with EGFP-positive HEK293 cells were performed the day after transfection with either the WT or the N1097Q mutant of TRPM7, using procedures described previously [45]. The standard extracellular solution contained in these experiments (in mM): 147 NaCl, 2 KCl, 1 MgCl_2_, 2 CaCl_2_, 13 d-glucose and 10 HEPES (∼305 mOsm/l; pH 7.3 with NaOH). A divalent cation-free (no-DIV) solution was produced by omitting Ca^2+^ and Mg^2+^ and supplementing EGTA and EDTA (1 mM each). Patch pipettes had a resistance of 15–20 MΩ when filled with intracellular solutions. These were either identical with the no-DIV saline or based on it but containing about 1 mM of free Mg^2+^ in addition (2.07 mM added MgCl_2_). Baselines in the current traces were corrected and raw single-channel data was evaluated with the QuB program. The mean amplitudes (*i*) of TRPM7 single-channel currents (digitized at 20 kHz and filtered at 2 kHz) were thereby from all-points amplitude histograms fitted to a sum of multiple Gaussian distributions, with the number of components depending on the apparent number of active channels (*N*) in a given patch. The probability for *N* channels being open (*NP*_O_) was, in turn, defined as the ratio of the area occupied by all the peaks for open channels to the total amplitude histogram (summation of the open and closed peaks). The unitary conductance (*γ*) of WT or mutant TRPM7 channels was either calculated from *i* as chord conductance (*γ*_c_), assuming a reversal potential of zero mV or, for comparison, in some experiments also derived from the slope of respective current–voltage relationships (*γ*_s_, slope conductance). In multichannel patches, like those usually obtained during this study, uncertainties concerning the “true” value of *N*, as well as the overlap of channel openings, precludes a detailed dwell time analysis.

To still allow for comparisons, such as between WT and N1097Q channels, the relation [53]:

$$T_{{{\text{os}}}} = \left( {\sum\limits_{i} \; L_{i} \;t_{i} } \right)/\;\# O,$$

where *t*_*i*_ is the total time the outside-out currents dwelt on level *L*_*i*_ during the recording and *#O* is the total of the number of opening events, was used to estimate a surrogate mean open time (*T*_OS_). To this end, channel openings were counted over a prolonged time (4 min) in the outside-out current recordings, using the 50% amplitude threshold criterion implemented in QuB. The dead time imposed was 200 μs (∼1.2-fold of the filter rise time), thus excluding shorter events than this from the analysis.

Data are presented as means ± standard error of the mean (means ± SEM). Statistical comparisons (Prism 8.4.0 or SigmaPlot 14.0) were made using analysis of variance (ordinary one-way ANOVA) or two-tailed *t *test, as indicated in the figure legends. Significance was accepted at *P* ≤ 0.05.

### MD simulations

All MD simulations were carried out using the pmemd.cuda program of AMBER16 molecular dynamics package [54]. The Amber FF99SB–ILDN force field [55] for proteins was used for all simulations combined with Lipid14 model [56] for lipids and TIP3P model for water. All covalent bonds involving hydrogen atoms were constrained using SHAKE [57] to allow an integration time step of 2 fs. Langevin thermostat and Berendsen barostat were used to control temperature and pressure, respectively. All NPT simulations were carried out using anisotropic pressure scaling. Electrostatic interactions were calculated using particle mesh ewald (PME) method [58] as implemented in Amber, with a non-bonded cutoff distance of 8 Å. Periodic boundary conditions were applied in all directions. In order to maintain the integrity of the pore loop of the protein, backbone hydrogen bond distances in the pore helix (residues 1031–1043) were restrained between 2.6 Å and the initial value during all simulations. Post-processing of trajectories was carried out using CPPTRAJ [59] and VMD [60].

#### MD simulation setup

Residues 982–1123 (S4-S5 linker, S5 helix, pore loop, S6 helix, and TRP helix) of the cryo-EM structure of the closed TRPM7 channel (PDB: 5ZX5) was used as the starting structure for all simulations. The protein was embedded in a POPC lipid membrane and solvated in water using the CHARMM-GUI Membrane Builder [61]. Additional water molecules were added manually to solvate the ion channel pore. The system was prepared for simulations using the charmmlipid2amber.py script and the tleap program in AmberTools16 (www.ambermd.org). The protein N and C termini were capped with the neutral acetyl and amide groups, and the conserved disulfide bond between residues C1056 and C1066 was introduced. The system was neutralized with Na^+^ and Cl^−^ ions. The final system contained 568 protein residues, 159 lipid molecules, 13,585 water molecules and neutralizing ions.

#### MD simulations of the closed TRPM7 channel

The prepared TRPM7 closed channel system was equilibrated as follows. First, a short minimization (6000 steps) was performed to remove clashes in the system. The system was then heated from 0.1 to 100 K in NVT ensemble and 100 K to 300 K in NPT ensemble, over 250 ps. The protein heavy atoms were restrained at their initial positions with a harmonic force constant (*k*) of 10 kcal mol^−1^ Å^−2^ during the heating steps (residues 1050–1070 in the pore loop were not restrained in order to enforce the disulphide bond between C1056 and C1066). The system was equilibrated at 300 K in NPT ensemble for 35 ns while gradually decreasing restraints on the protein until only the *C*_α_ atoms were restrained with *k* = 0.5 kcal mol^−1^ Å^−2^. The resulting system was used as the starting structure for the simulations with Mg^2+^ and N1097 mutant simulations (described in the following sections). All restraints on protein *C*_α_ atoms were removed except those on residues 1116–1123 of the TRP helix, and the closed channel was equilibrated in NPT ensemble for further 100 ns.

#### TRPM7 open channel conformation model

The S5, S6, and TRP helices and the S4–S5 linker (residues 982–1022 and 1071–1123) of the TRPM7 open channel were modelled in SWISS-MODEL [62] using the open channel structure of human TRPV6 channel (PDB ID: 6BO8) as a template. The target–template alignment for homology modelling was extracted from a multiple sequence alignment of the transmembrane and TRP regions of known TRPM and TRPV structures (TRPM7, TRPM4, TRPM2, TRPV1, TRPV3, TRPV6) performed using Clustal Omega at EMBL-EBI [63]. Residues 471–513 and 553–605 of PDB: 6BO8 was used to model the open structure of TRPM7.

#### MD simulations of the open channel

The homology model of the open TRPM7 channel was used to open the equilibrated closed channel by targeted MD simulations with simulated annealing. To open the channel, the *C*_α_ atoms of residues 982–1022 and 1071–1123 (S4–S5 and S5 helix, S6 and TRP helices) in the closed channel were harmonically restrained to the corresponding coordinates of the open homology model. Simulated annealing was performed in six steps as follows. In each step, (a) the system was equilibrated for 5 ns at 300 K, (b) heated to 350 K over 500 ps, (c) equilibrated at 350 K for 5 ns (or until RMSD of the targeted *C*_α_ atoms with respect to the reference structure was stable), and (d) cooled back to 300 K over 500 ps. Harmonic restraints were maintained throughout the process, increasing the force constant at the end of each step from 0.05, 0.1, 0.2, 1.0, 2.0 to 2.5 kcal mol^−1^ Å^−2^. The restraints were then released gradually over a period of 25 ns and, finally, unrestrained simulations of the open model were carried out for over 200 ns.

#### MD simulations of the mutant TRPM7 variants

The partially equilibrated closed TRPM7 channel (see “MD simulations of the closed TRPM7 channel” section) was used to construct the mutants N1097Q, N1097A and N1098Q. Additionally, Mg^2+^ was placed between the residues at the 1097 position to obtain complexes of the wild type (WT-MG), and the N1097Q (N1097Q-MG) and N1098Q (N1098Q-MG) mutants with Mg^2+^. The charge on Mg^2+^ was set to + 1.65. Each system was equilibrated using a protocol similar to that described in the previous section (MD simulations of the closed TRPM7 channel), except restraints on the protein *C*_α_ atoms were changed from *k* = 10 to 0.5 kcal mol^−1^ Å^−2^ in 20 ns. In WT-MG, N1097Q-MG and N1098Q-MG systems, the magnesium ion was restrained with the same force constant used to restrain the protein, and an additional equilibration step of 100 ns was carried out without restraints on the magnesium ion while maintaining restraints on the protein *C*_α_ atoms at *k *= 0.5 kcal mol^−1^ Å^−2^. All systems were simulated for 100 ns with all restraints removed except the restraints on *C*_α_ atoms of residues 1116–1123.

### Supplementary Information

Below is the link to the electronic supplementary material.

**Suppl. Figure S1**. Multiple sequence alignment of amino-acid sequences encoding the S2, S3 and TRP segments in the mouse TRPM1–8 proteins. Conserved E, D, N and Q residues forming Ca^2+^-binding pockets in TRPM2, TRPM4, TRPM5 and TRPM8 are labelled in green or blue and highlighted in grey. Note that these residues are only partially retained in TRPM7 as indicated accordingly. (PDF 145 KB)

**Suppl. Figure S2.** Subcellular localization of TRPM7 in HEK293T cells. Mouse TRPM7 variants were transiently expressed in the indicated TRPM7 cDNA plasmid variants in HEK293T cells and immunolocalized using anti-TRPM7 and anti-mouse IgG-Alexa Fluor 488 antibodies. Representative confocal images of Alexa Fluor 488 fluorescence (Left panels) and their overlay with corresponding DIC images (Middle and Right panels) are shown. Scale bars are 5 μm. (PDF 183 KB)

**Suppl. Figure S3.** Inhibition of TRPM7 currents by intracellular Ba^2+^. Whole-cell currents were measured in HEK293T cells transfected by WT (**A**), N1097Q (**B**) and N1098Q (**C**) variants of TRPM7 cDNAs (in pIRES2-EGFP). Left panels: Current amplitudes (mean ± SEM) were measured at +80 mV and plotted over time. Currents were measured using an intracellular solution containing the standard [Mg^2+^]_i_-free intracellular solution and solutions containing 0.55 and 1 mM free [Ba^2+^]_i_ (Suppl. Table S2). Middle panels: Representative I-V relationships obtained from individual ramps at 300 s in the Left panels. Right panels: Bar graphs of outward currents (+80 mV, mean ± SEM) obtained at 300 s as indicated in the Left panels. n, number of cells measured; n.s., not significant; **P< 0.01, *** P< 0.001 (ANOVA) (PDF 355 KB)

**Suppl. Figure S4.** Inhibition of TRPM7 currents by cytosolic Mg·ATP and Mg·GTP. Whole-cell currents were measured in HEK293T cells transfected by WT (**A**,** B**), N1097Q (**C**,** D**) and N1098Q (**E**,** F**) variants of TRPM7 cDNAs (in pIRES2-EGFP). A, C, D Current amplitudes (mean ± SEM) were measured at +80 mV and plotted over time. Currents were measured using an intracellular solution containing 250 µM free [Mg^2+^]_i_ without Mg·nucleotides, 250 µM free [Mg^2+^]_i_ with 6 mM [Mg·ATP]_i_, and 250 µM free [Mg^2+^]_i_ with 6 mM [Mg·GTP]_i_ (Suppl. Table S3). B, D, F Bar graphs of outward currents (+80 mV, mean ± SEM) obtained at 300 s as indicated in (A, B, C). Note: The results obtained with 250 µM free [Mg^2+^]_i_ without Mg·nucleotides and with 6 mM [Mg·ATP]_i_ were taken from Fig. 3. n, number of cells measured; n.s., not significant; ***P< 0.001 (ANOVA) (PDF 216 KB)

**Suppl. Figure S5.** Suppression of TRPM7 currents by 9 mM Mg·ATP in the presence of 1 mM free Mg^2+^. Whole-cell currents were measured and analysed analogously to the experiment outlined in Figure 3G, except that 9 mM [Mg·ATP]_i_ and 1 mM free [Mg^2+^]_i_ were included in the intracellular solution (Suppl. Table S4). n, number of cells measured; n.s., not significant; * P< 0.05, ** P< 0.01 (ANOVA). (PDF 61 KB)

**Suppl. Figure S6.** Examination of TRPM7 currents in the perforated patch.Current-voltage (I-V) relationships of currents were measured in HEK293T cells transfected by WT (**A**), N1097Q (**B**) and N1098Q (**C**) variants of TRPM7 cDNAs. The I-V relationships were acquired after 25 s of breake-in using the standard intracellular solution containing 320 µM amphotericin B.** D** Bar graphs of outward currents (+80 mV, 25 s) shown in (A–C). n, number of cells measured; **P< 0.01 (ANOVA) (PDF 49 KB)

**Suppl. Figure S7.** Assessment of TRPM7 currents using a divalent cation-free (DVF) extracellular solution.Whole-cell currents of WT (**A**), N1097Q (**B**) and N1098Q (**C**) TRPM7 variants (in pIRES2-EGFP) expressed in HEK293T cells. Left panels: Current amplitudes (mean ± SEM) were measured at − 80 and + 80 mV and plotted over time. Currents were induced using the standard [Mg^2+^]_i_ free intracellular solution and the standard external solution. When currents were fully activated, cells were perfused with the DVF solution as indicated by the black bars. Right panels: Representative I-V relationships obtained from individual ramps before (brown) and after (red) DVF application as indicated in the Left panels by coloured data points. D Bar graphs of outward (+ 80 mV; Left panel) and inward (− 80 mV; Right panel) currents (mean ± SEM) shown in (A–C) at 200 s. n, number of cells measured; n.s., not significant (ANOVA). (PDF 230 KB)

**Suppl. Figure S8.** Single-channel properties of the WT and N1097Q TRPM7 variants. Currents were recorded in outside-out membrane patches excised from HEK293 cells expressing WT (**A**,** C**) and N1097Q (**B**,** C**) variants of TRPM7. The intracellular solution was no-DIV (0 mM Mg^2+^). A, B Shown are current traces at a holding potential of − 60 mV from WT (A) and N1097Q channels (B), starting (from top to bottom) 2 s before channels were unblocked by switching from a divalent cation-containing bath solution (DIV) to a divalent-free bath (no-DIV) and 28 s and 238 s after solution exchange. C The left panel shows representative current traces from the same patches as in (A) and (B), obtained at the indicated patch potentials in a no-DIV bath. The right panel shows a plot of unitary current amplitudes versus patch potential constructed thereof, revealing a linear I-V relationships. The slope conductance (*γs*) derived from linear regression fitting (R2 = 0.997 and 0.997) was in these two outside-out patches 44 pS and 21 pS for WT and N1097Q channels, respectively. Insets: Statistical evaluation of outside-out recordings with the WT channel and N1097Q in the absence of intracellular Mg^2+^. Note that the open probability (*NP*_*O*_) was similar for the TRPM7 variants (*P* = 0.806), despite the significantly shorter open time (*T*_*OS*_) of N1097Q (*n* = 7 cells,* P* = 0.002). The NP_O_ and *T*_*OS*_ data for N1097Q are taken from Fig. 7 and replotted here for comparison (PDF 524 KB)

**Suppl. Figure S9.** Impact of intracellular Mg^2+^ and Mg·ATP on single-channel TRPM7 currents. Currents were recorded at a holding potential of – 60 mV in outside-out membrane patches excised from HEK293 cells expressing WT (**A**,** B**) and N1097Q (**C**–**E**) variants of TRPM7. The WT (A) or N1097Q (C) channels were induced by removing the extracellular divalent cations (DIV bath) using a no-DIV solution, as indicated above the current traces. The intracellular solution was no-DIV containing 3 mM Mg·ATP and 0.5 mM MgCl_2_, resulting in 2.9 mM [Mg·ATP]_i_ and 250 μM free [Mg^2+^]_i_. Insets: currents on an expanded time scale from the segments indicated by arrows. The graphs on the right show all-point histograms from the two 30 s current traces. The dotted line indicates the closed level (*C*). The broken lines indicate the current level for 1 channel (*O1*), 2 channels (*O2*) or 3 channels (*O3*) being open. Single-channel amplitudes (i) taken from O1 were -2.4 pA and -1.4 pA for WT channels (A) and N1097Q (C), respectively. B, D The open probabilities (NPO) assessed for bins of 2 s over the whole 4.5 min duration of the experiments shown in (A, C). E Statistical evaluation of outside-out recordings with the N1097Q channel. The data obtained with 0 and 1 mM Mg^2+^ were replotted from Fig. 7. Note that intracellular Mg·ATP did not affect single-channel chord conductance (γc; P = 0.43, one-way ANOVA), NP_O_ (P = 0.91, one-way ANOVA) and open time (*T*_*OS*_;* n* = 3–7;* P* = 0.925, one-way ANOVA) (PDF 175 KB)

**Suppl. Figure S10**. Homology modelling of the S4-S5, S5, S6, and TRP domains of the open TRPM7 channel using the TRPV6 open channel as a template.** A** Sequence alignment of residues 982-1022 and 1071-1123 of TRPM7, and residues 471 to 513 and 553 to 605 of the human TRPV6 open channel (PDB 6BO8).** B** Overlap of the homology model (green) and the cryo-EM structure of the closed TRPM7 (PDB 6BWD, blue) (PDF 97 KB)

**Suppl. Figure S11.** The proposed arrangements of N1097 and N1098 in the lower channel gate of TRPM7. **A** Comparison of the interaction of Mg^2+^ at the lower gate in simulated WT and N1097Q channels. Protein residues that interact with Mg^2+^ are shown in yellow. Water molecules (WAT) that interact with the Mg^2+^ ion are shown as red and white spheres.** B** Representative snapshots of WT and N1098Q channels displaying hydrogen bond interactions (dashed lines) of residue 1098 (yellow) viewed intracellularly. N1097 (blue) and other residues that interact with the mutated residue in at least one subunit (white) are shown. Residues in diagonal subunit pairs are labelled in different colours. Note that the hydrogen bonds are transient, and the specific interactions shown are not present in all frames of the trajectory (PDF 208 KB)

Supplementary file12 (DOCX 18 KB)

## Data Availability

All reagents and data generated or analysed during this study and its supplementary information files are available from the corresponding authors on request.
