# Supplementary material for: Structural mechanism of TRPM7 channel regulation by intracellular magnesium
Source: Cell Mol Life Sci. 2022 Apr 7;79(5):225. doi: 10.1007/s00018-022-04192-7 (PMC8989868; doi:10.1007/s00018-022-04192-7)
Supplement: Supplementary file 1 — Suppl. Figure S1. Multiple sequence alignment of amino-acid sequences encoding the S2, S3 and TRP segments in the mouse TRPM1–8 proteins. Conserved E, D, N and Q residues forming Ca2+-binding pockets in TRPM2, TRPM4, TRPM5 and TRPM8 are labelled in green or blue and highlighted in grey. Note that these residues are only partially retained in TRPM7 as indicated accordingly. (PDF 145 KB) [file 18_2022_4192_MOESM1_ESM.pdf]

Suppl. Figure S1

```
                  S2                                                     S3
TRPM7   896 YAIEKVREVFMSE------------AGKISQKIKVWFSDYFNVSDTIAI 932
TRPM6   888 NAIEKVREICISE------------PSKFKQKVKMWLSEYWNLMETVAI 924
TRPM1   916 LALEKIREILMSE------------PGKLSQKIKVWLQEYWNITDLVAI 952
TRPM3   924 LGIEKMREILMSE------------PGKLLQKVKVWLQEYWNVTDLIAI 960
TRPM2   836 LVCEETRQLFYD-----------PDGCGLMKMASLYFSDFWNKLDVGAI 873
TRPM4   820 LLCEELRQGLGGGWGSLASGGRGPDRAPLRHRLHLYLSDTWNQCDLLAL 868
TRPM5   777 LVLEEIRQGFFT-----------DEDTHLVKKFTLYVEDNWNKCDMVAI 814
TRPM8   778 LFCDEVRQWYMNG--------------------VNYFTDLWNVMDTLGL 806

                     TRP
TRPM7  1106 ISNIVWKYQRYHFIMAYHEK 1125
TRPM6  1085 ISNKLWKYNRYRYIMTYHQK 1104
TRPM1  1130 ISNQVWKFQRYQLIMTFHDR 1149
TRPM3  1133 ISNQVWKFQRYQLIMTFHER 1152
TRPM2  1055 HTDQIWKFQRHDLIEEYHGR 1068
TRPM4  1049 NSDLYWKAQRYSLIREFHSR 1097
TRPM5   987 NADMFWKFQRYHLIVEYHGR 1006
TRPM8   989 NNDQVWKFQRYFLVQEYCNR 1008
```
