# Supplementary figures and images for: Structural mechanism of TRPM7 channel regulation by intracellular magnesium
Source: Cell Mol Life Sci. 2022 Apr 7;79(5):225. doi: 10.1007/s00018-022-04192-7 (PMC8989868; doi:10.1007/s00018-022-04192-7)

Suppl. Figure S2

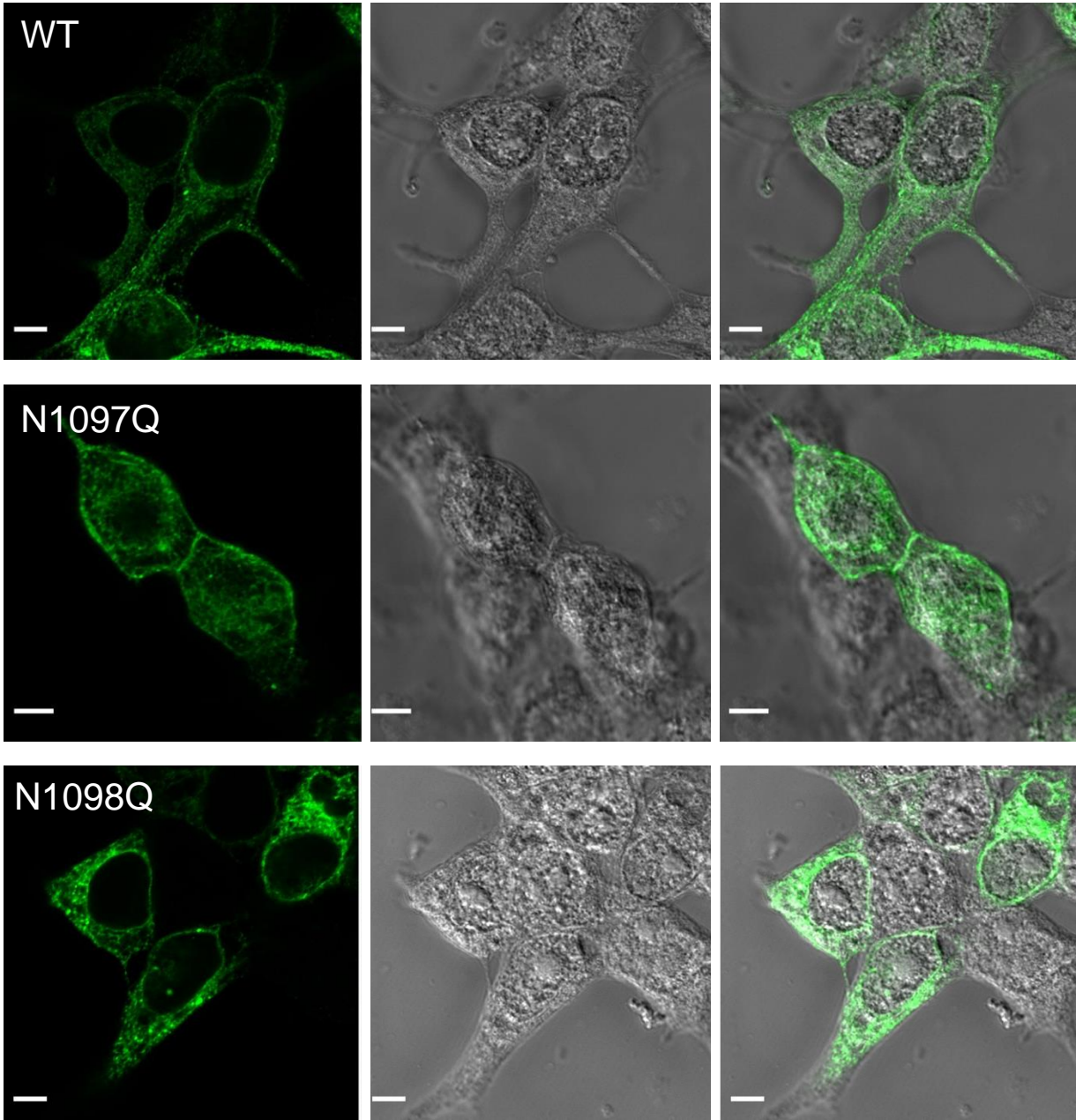

Supplement: Supplementary file 2 — Suppl. Figure S2. Subcellular localization of TRPM7 in HEK293T cells. Mouse TRPM7 variants were transiently expressed in the indicated TRPM7 cDNA plasmid variants in HEK293T cells and immunolocalized using anti-TRPM7 and anti-mouse IgG-Alexa Fluor 488 antibodies. Representative confocal images of Alexa Fluor 488 fluorescence (Left panels) and their overlay with corresponding DIC images (Middle and Right panels) are shown. Scale bars are 5 μm. (PDF 183 KB) [file 18_2022_4192_MOESM2_ESM.pdf]

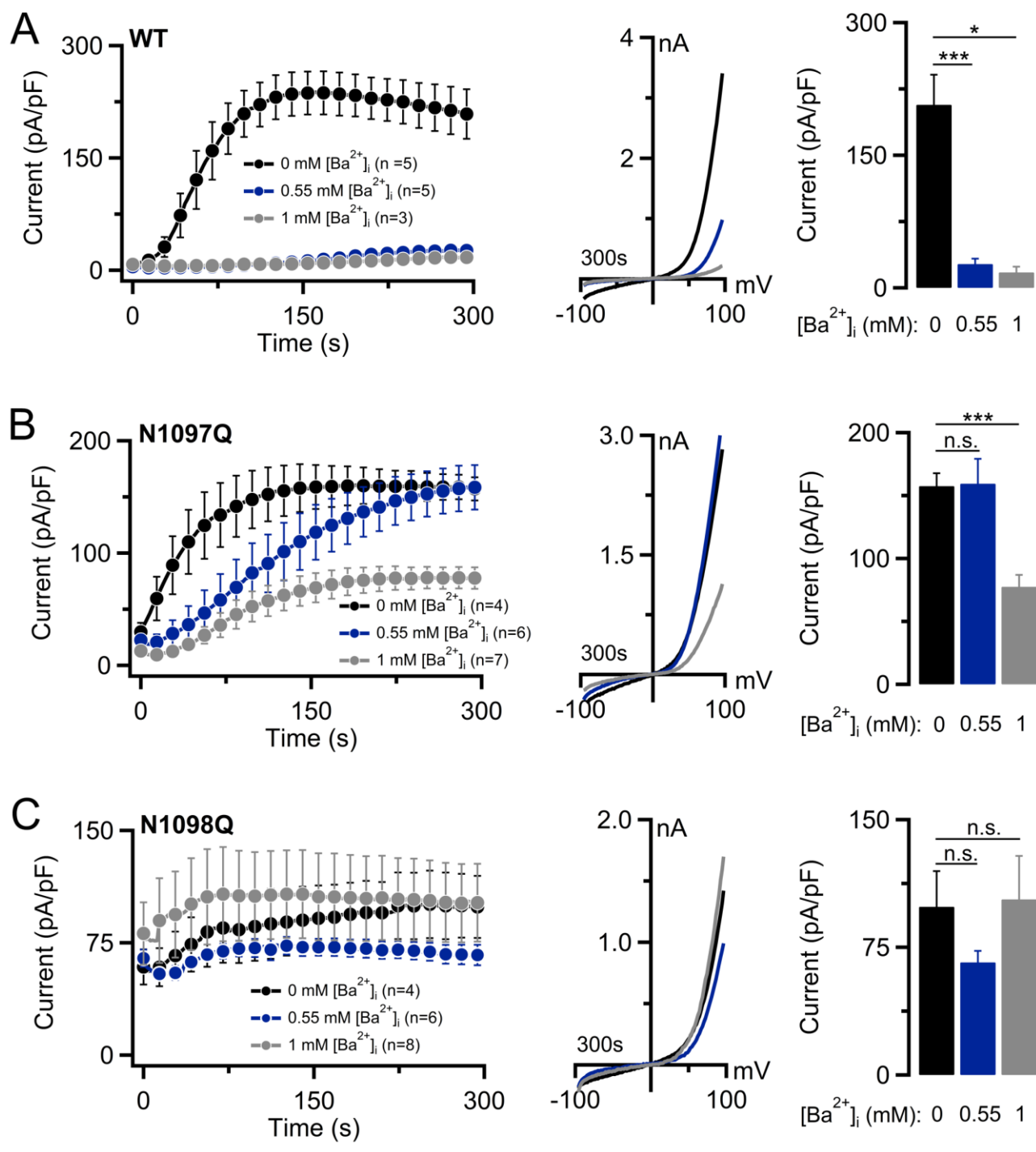

Supplement: Supplementary file 3 — Suppl. Figure S3. Inhibition of TRPM7 currents by intracellular Ba2+. Whole-cell currents were measured in HEK293T cells transfected by WT (A), N1097Q (B) and N1098Q (C) variants of TRPM7 cDNAs (in pIRES2-EGFP). Left panels: Current amplitudes (mean ± SEM) were measured at +80 mV and plotted over time. Currents were measured using an intracellular solution containing the standard [Mg2+]i-free intracellular solution and solutions containing 0.55 and 1 mM free [Ba2+]i (Suppl. Table S2). Middle panels: Representative I-V relationships obtained from individual ramps at 300 s in the Left panels. Right panels: Bar graphs of outward currents (+80 mV, mean ± SEM) obtained at 300 s as indicated in the Left panels. n, number of cells measured; n.s., not significant; **P< 0.01, *** P< 0.001 (ANOVA) (PDF 355 KB) [file 18_2022_4192_MOESM3_ESM.pdf]

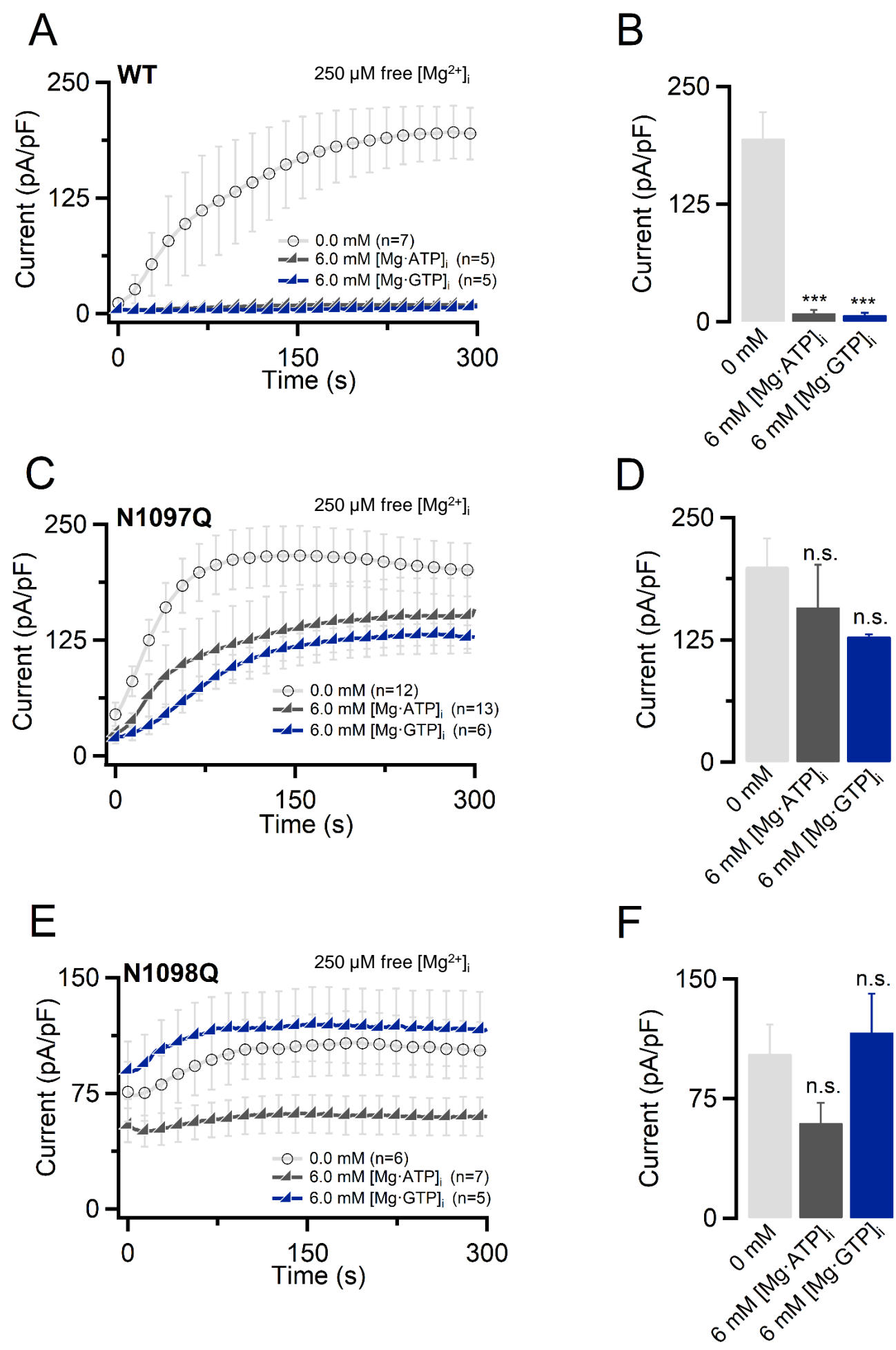

Supplement: Supplementary file 4 — Suppl. Figure S4. Inhibition of TRPM7 currents by cytosolic Mg·ATP and Mg·GTP. Whole-cell currents were measured in HEK293T cells transfected by WT (A, B), N1097Q (C, D) and N1098Q (E, F) variants of TRPM7 cDNAs (in pIRES2-EGFP). A, C, D Current amplitudes (mean ± SEM) were measured at +80 mV and plotted over time. Currents were measured using an intracellular solution containing 250 µM free [Mg2+]i without Mg·nucleotides, 250 µM free [Mg2+]i with 6 mM [Mg·ATP]i, and 250 µM free [Mg2+]i with 6 mM [Mg·GTP]i (Suppl. Table S3). B, D, F Bar graphs of outward currents (+80 mV, mean ± SEM) obtained at 300 s as indicated in (A, B, C). Note: The results obtained with 250 µM free [Mg2+]i without Mg·nucleotides and with 6 mM [Mg·ATP]i were taken from Fig. 3. n, number of cells measured; n.s., not significant; ***P< 0.001 (ANOVA) (PDF 216 KB) [file 18_2022_4192_MOESM4_ESM.pdf]

Suppl. Figure S5

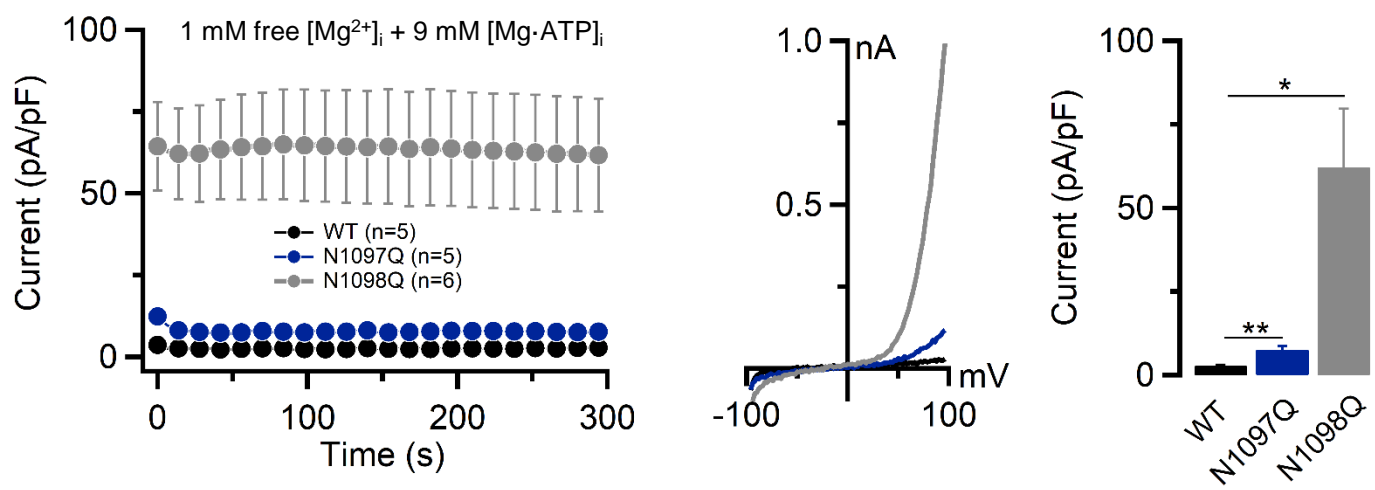

Supplement: Supplementary file 5 — Suppl. Figure S5. Suppression of TRPM7 currents by 9 mM Mg·ATP in the presence of 1 mM free Mg2+. Whole-cell currents were measured and analysed analogously to the experiment outlined in Figure 3G, except that 9 mM [Mg·ATP]i and 1 mM free [Mg2+]i were included in the intracellular solution (Suppl. Table S4). n, number of cells measured; n.s., not significant; * P< 0.05, ** P< 0.01 (ANOVA). (PDF 61 KB) [file 18_2022_4192_MOESM5_ESM.pdf]

Suppl. Figure S6

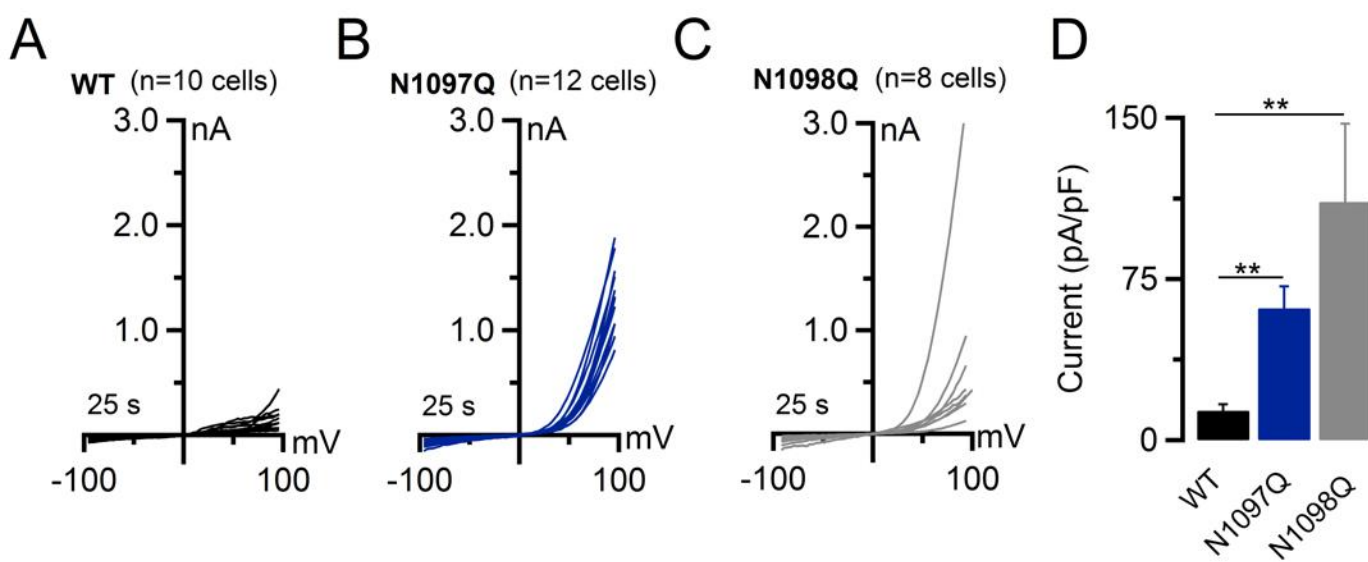

Supplement: Supplementary file 6 — Suppl. Figure S6. Examination of TRPM7 currents in the perforated patch.Current-voltage (I-V) relationships of currents were measured in HEK293T cells transfected by WT (A), N1097Q (B) and N1098Q (C) variants of TRPM7 cDNAs. The I-V relationships were acquired after 25 s of breake-in using the standard intracellular solution containing 320 µM amphotericin B. D Bar graphs of outward currents (+80 mV, 25 s) shown in (A–C). n, number of cells measured; **P< 0.01 (ANOVA) (PDF 49 KB) [file 18_2022_4192_MOESM6_ESM.pdf]

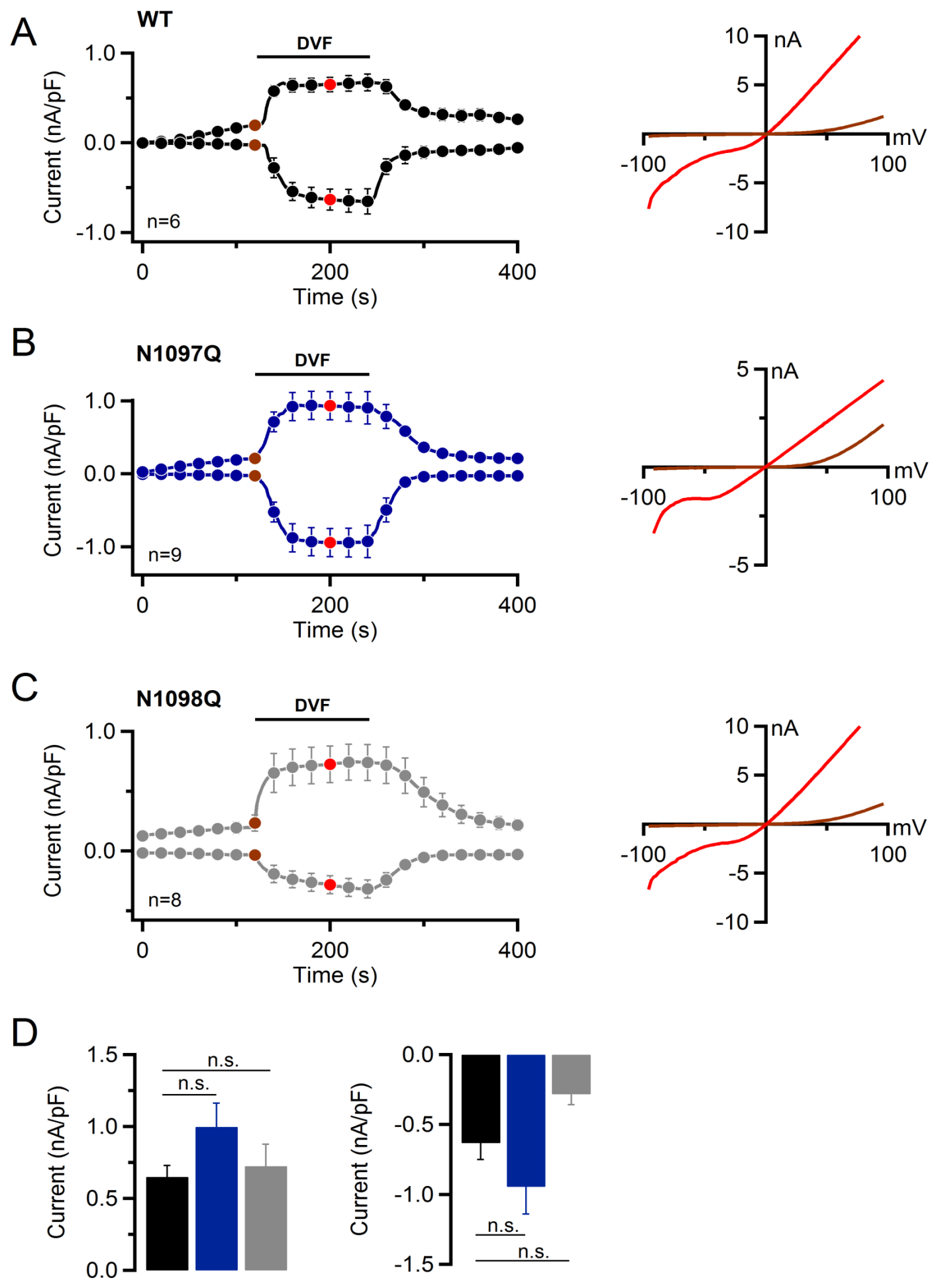

Supplement: Supplementary file 7 — Suppl. Figure S7. Assessment of TRPM7 currents using a divalent cation-free (DVF) extracellular solution.Whole-cell currents of WT (A), N1097Q (B) and N1098Q (C) TRPM7 variants (in pIRES2-EGFP) expressed in HEK293T cells. Left panels: Current amplitudes (mean ± SEM) were measured at − 80 and + 80 mV and plotted over time. Currents were induced using the standard [Mg2+]i free intracellular solution and the standard external solution. When currents were fully activated, cells were perfused with the DVF solution as indicated by the black bars. Right panels: Representative I-V relationships obtained from individual ramps before (brown) and after (red) DVF application as indicated in the Left panels by coloured data points. D Bar graphs of outward (+ 80 mV; Left panel) and inward (− 80 mV; Right panel) currents (mean ± SEM) shown in (A–C) at 200 s. n, number of cells measured; n.s., not significant (ANOVA). (PDF 230 KB) [file 18_2022_4192_MOESM7_ESM.pdf]

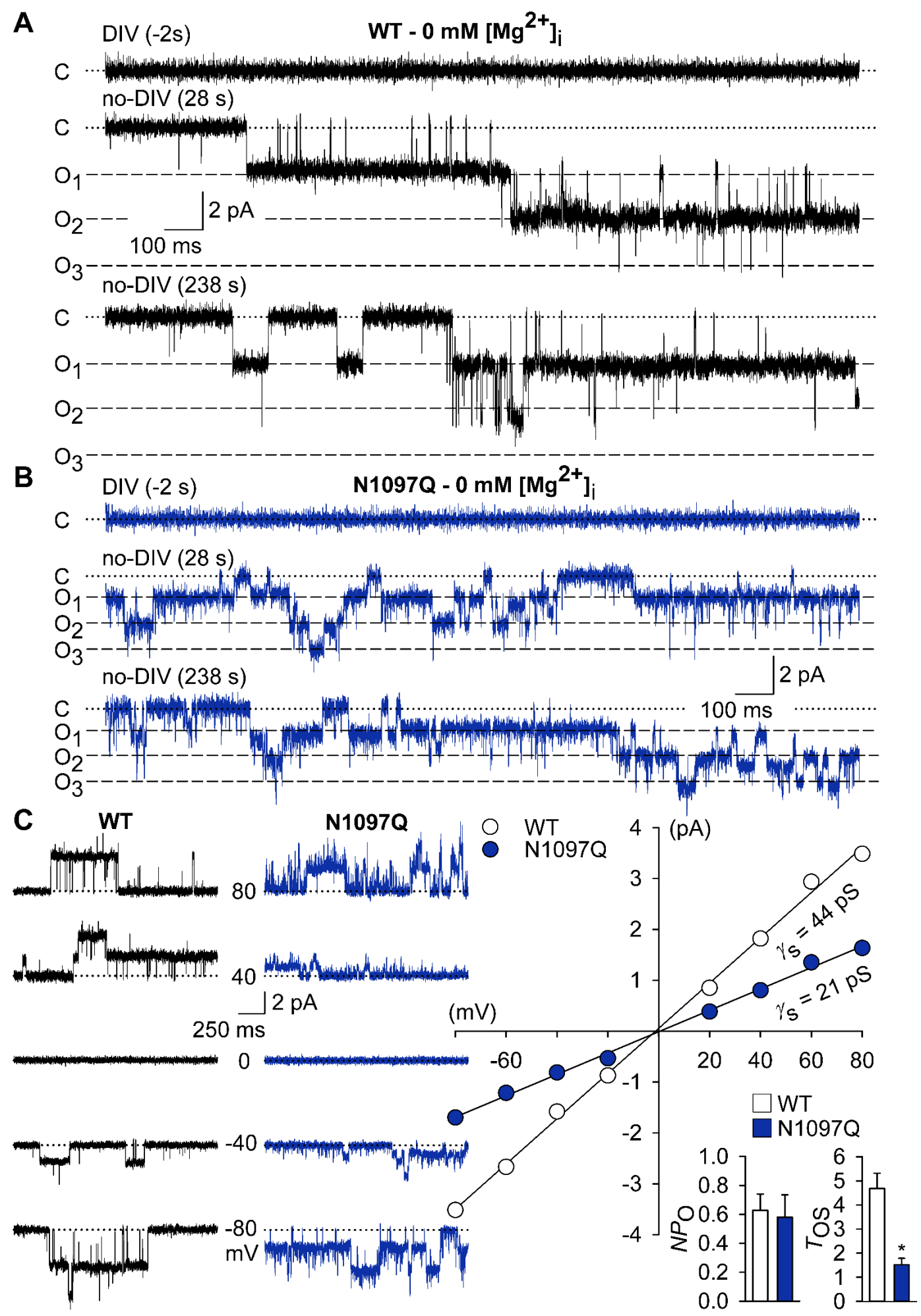

Supplement: Supplementary file 8 — Suppl. Figure S8. Single-channel properties of the WT and N1097Q TRPM7 variants. Currents were recorded in outside-out membrane patches excised from HEK293 cells expressing WT (A, C) and N1097Q (B, C) variants of TRPM7. The intracellular solution was no-DIV (0 mM Mg2+). A, B Shown are current traces at a holding potential of − 60 mV from WT (A) and N1097Q channels (B), starting (from top to bottom) 2 s before channels were unblocked by switching from a divalent cation-containing bath solution (DIV) to a divalent-free bath (no-DIV) and 28 s and 238 s after solution exchange. C The left panel shows representative current traces from the same patches as in (A) and (B), obtained at the indicated patch potentials in a no-DIV bath. The right panel shows a plot of unitary current amplitudes versus patch potential constructed thereof, revealing a linear I-V relationships. The slope conductance (γs) derived from linear regression fitting (R2 = 0.997 and 0.997) was in these two outside-out patches 44 pS and 21 pS for WT and N1097Q channels, respectively. Insets: Statistical evaluation of outside-out recordings with the WT channel and N1097Q in the absence of intracellular Mg2+. Note that the open probability (NPO) was similar for the TRPM7 variants (P = 0.806), despite the significantly shorter open time (TOS) of N1097Q (n = 7 cells, P = 0.002). The NPO and TOS data for N1097Q are taken from Fig. 7 and replotted here for comparison (PDF 524 KB) [file 18_2022_4192_MOESM8_ESM.pdf]

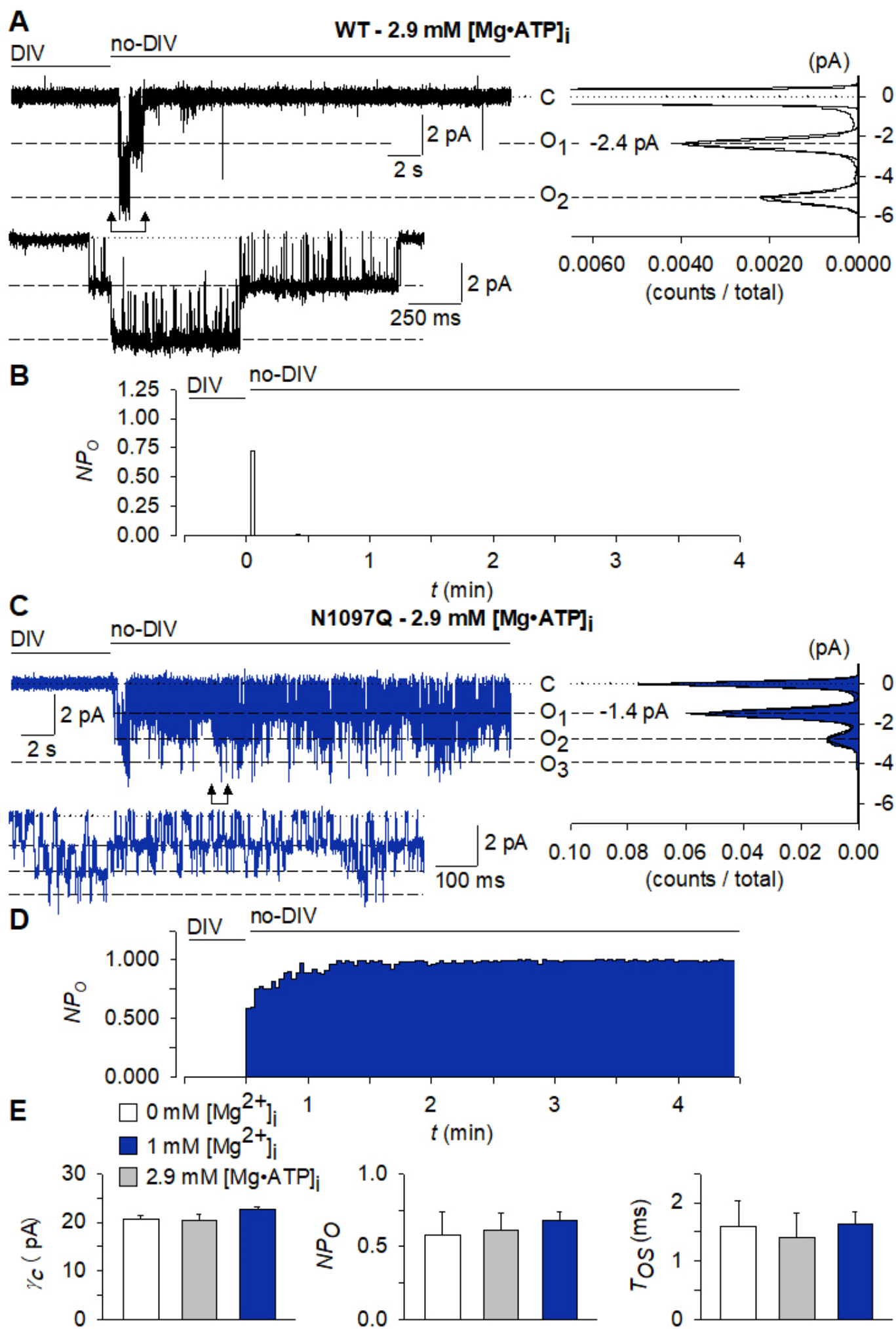

Supplement: Supplementary file 9 — Suppl. Figure S9. Impact of intracellular Mg2+ and Mg·ATP on single-channel TRPM7 currents. Currents were recorded at a holding potential of – 60 mV in outside-out membrane patches excised from HEK293 cells expressing WT (A, B) and N1097Q (C–E) variants of TRPM7. The WT (A) or N1097Q (C) channels were induced by removing the extracellular divalent cations (DIV bath) using a no-DIV solution, as indicated above the current traces. The intracellular solution was no-DIV containing 3 mM Mg·ATP and 0.5 mM MgCl2, resulting in 2.9 mM [Mg·ATP]i and 250 μM free [Mg2+]i. Insets: currents on an expanded time scale from the segments indicated by arrows. The graphs on the right show all-point histograms from the two 30 s current traces. The dotted line indicates the closed level (C). The broken lines indicate the current level for 1 channel (O1), 2 channels (O2) or 3 channels (O3) being open. Single-channel amplitudes (i) taken from O1 were -2.4 pA and -1.4 pA for WT channels (A) and N1097Q (C), respectively. B, D The open probabilities (NPO) assessed for bins of 2 s over the whole 4.5 min duration of the experiments shown in (A, C). E Statistical evaluation of outside-out recordings with the N1097Q channel. The data obtained with 0 and 1 mM Mg2+ were replotted from Fig. 7. Note that intracellular Mg·ATP did not affect single-channel chord conductance (γc; P = 0.43, one-way ANOVA), NPO (P = 0.91, one-way ANOVA) and open time (TOS; n = 3–7; P = 0.925, one-way ANOVA) (PDF 175 KB) [file 18_2022_4192_MOESM9_ESM.pdf]

A

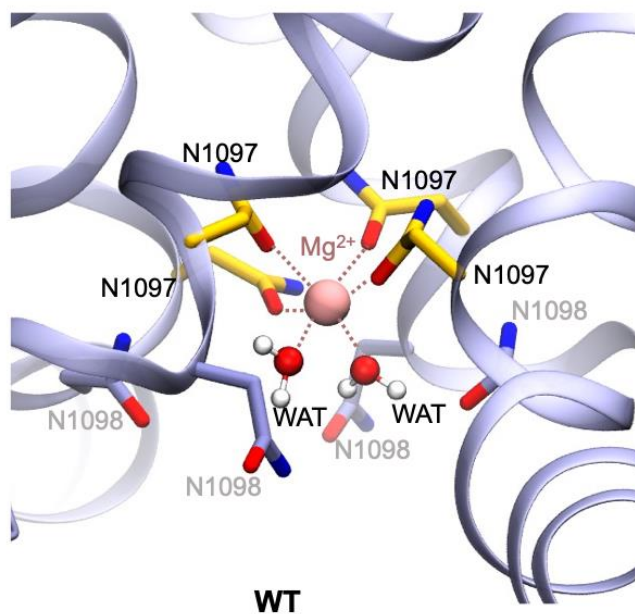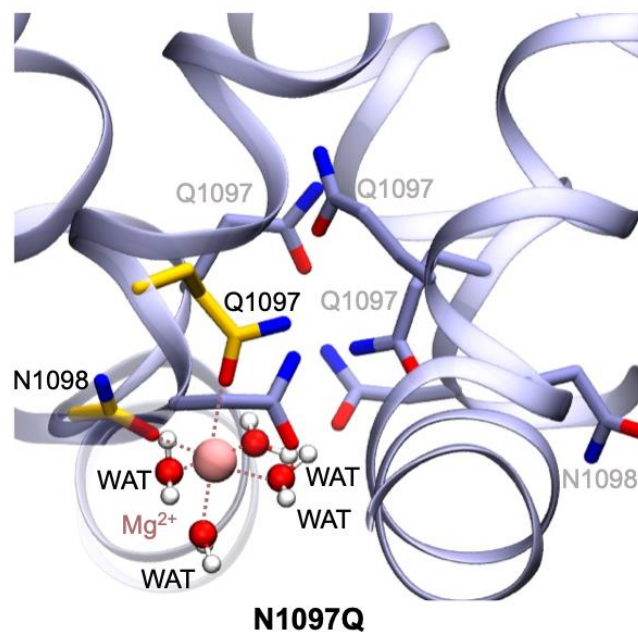

B

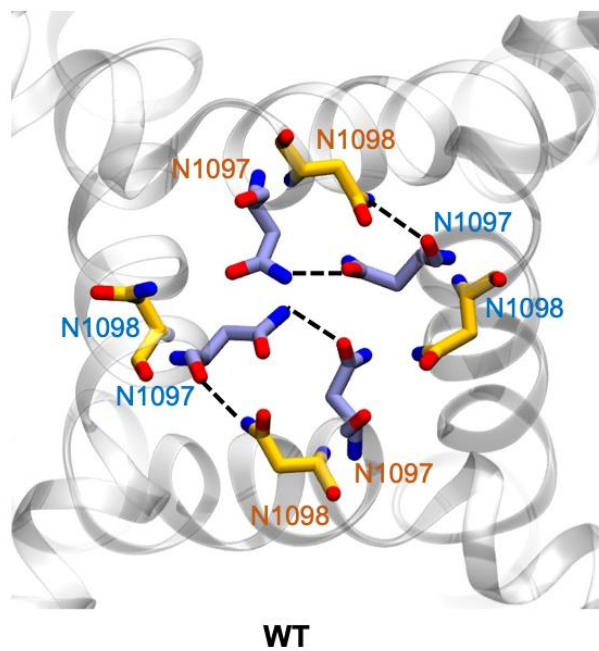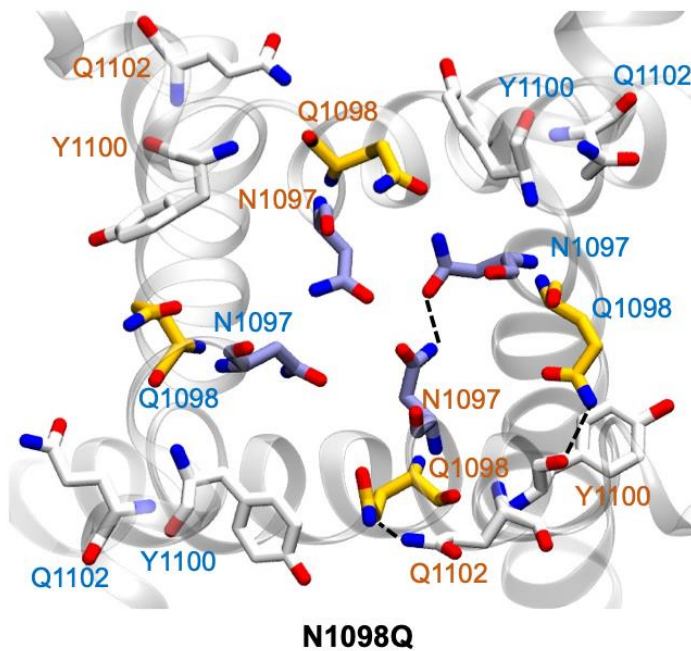

Supplement: Supplementary file 11 — Suppl. Figure S11. The proposed arrangements of N1097 and N1098 in the lower channel gate of TRPM7. A Comparison of the interaction of Mg2+ at the lower gate in simulated WT and N1097Q channels. Protein residues that interact with Mg2+ are shown in yellow. Water molecules (WAT) that interact with the Mg2+ ion are shown as red and white spheres. B Representative snapshots of WT and N1098Q channels displaying hydrogen bond interactions (dashed lines) of residue 1098 (yellow) viewed intracellularly. N1097 (blue) and other residues that interact with the mutated residue in at least one subunit (white) are shown. Residues in diagonal subunit pairs are labelled in different colours. Note that the hydrogen bonds are transient, and the specific interactions shown are not present in all frames of the trajectory (PDF 208 KB) [file 18_2022_4192_MOESM11_ESM.pdf]
