# Supplementary material for: Structural mechanism of TRPM7 channel regulation by intracellular magnesium
Source: Cell Mol Life Sci. 2022 Apr 7;79(5):225. doi: 10.1007/s00018-022-04192-7 (PMC8989868; doi:10.1007/s00018-022-04192-7)

Suppl. Figure S10

A

```
TRPM7   982  V N Q Q A G P Y V M M I G K M V A - N M F Y I V V I M A L V L L S F G V P R K A I L Y  1022
TRPV6   471  G F Q M L G P F T I M I Q K M I F G D L M R F C W L M A V V I L G F A S A F Y I I F Q  513

TRPM7  1071  W L T P F L Q A V Y L F V Q Y I I M V N L L I A F F N N V Y L Q V K A I S N I V W K Y  1113
TRPV6   553  F M Y S I T Y A A F A I I A T L L M L N L L I A M M G D T H W R V A H E R D E L W R A  595

TRPM7  1114  Q R Y H F I M A Y H  1123
TRPV6   596  Q I V A T T V M L E  605
```

B

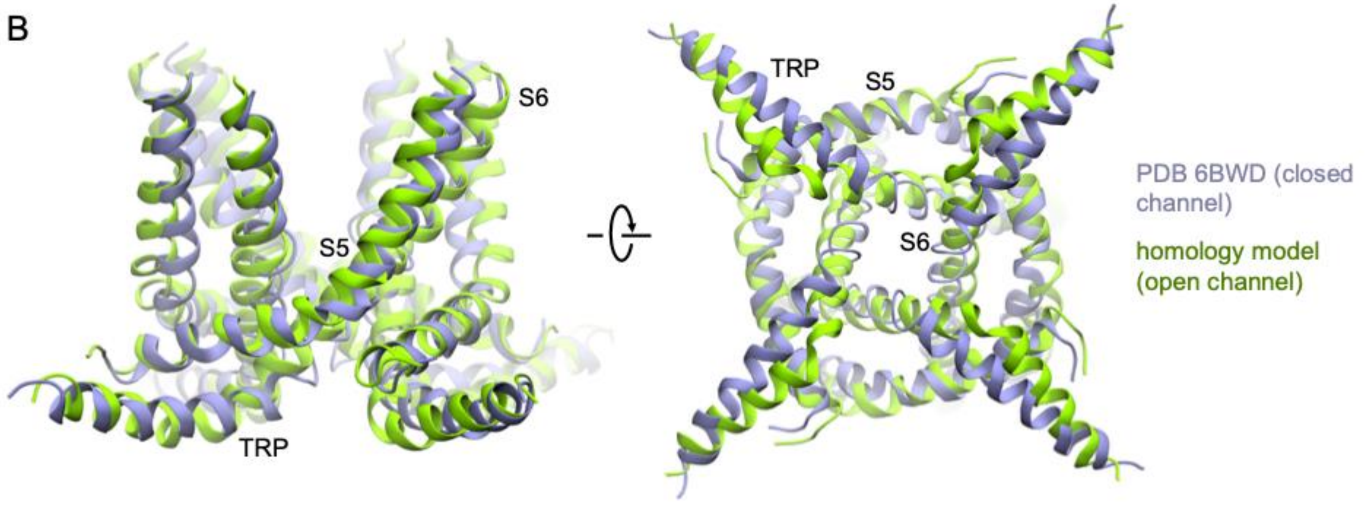

Supplement: Supplementary file 10 — Suppl. Figure S10. Homology modelling of the S4-S5, S5, S6, and TRP domains of the open TRPM7 channel using the TRPV6 open channel as a template. A Sequence alignment of residues 982-1022 and 1071-1123 of TRPM7, and residues 471 to 513 and 553 to 605 of the human TRPV6 open channel (PDB 6BO8). B Overlap of the homology model (green) and the cryo-EM structure of the closed TRPM7 (PDB 6BWD, blue) (PDF 97 KB) [file 18_2022_4192_MOESM10_ESM.pdf]
