# Supplementary material for: Structural mechanism of TRPM7 channel regulation by intracellular magnesium
Source: Cell Mol Life Sci. 2022 Apr 7;79(5):225. doi: 10.1007/s00018-022-04192-7 (PMC8989868; doi:10.1007/s00018-022-04192-7)
Supplement: Supplementary file 12 — Supplementary file12 (DOCX 18 KB) [file 18_2022_4192_MOESM12_ESM.docx]

**Suppl. Table S1.** Internal solutions used to study Mg^2+^ concentration-dependent inhibition of TRPM7 currents.

| ^a^Free [Mg^2+^]_i_  (mM) | Cs-glutamate  (mM) | NaCl  (mM) | Cs-HEPES  (mM) | Cs-EGTA  (mM) | MgCl_2_  (mM) |
| --- | --- | --- | --- | --- | --- |
| 0.10 | 140 | 8 | 10 | 10 | 0.16 |
| 0.25 | 120 | 8 | 10 | 10 | 0.38 |
| 0.55 | 120 | 8 | 10 | 10 | 0.83 |
| 1.00 | 120 | 8 | 10 | 10 | 1.50 |
| 2.30 | 120 | 8 | 10 | 10 | 3.40 |
| 5.00 | 120 | 8 | 10 | 10 | 7.10 |
| 7.00 | 120 | 8 | 10 | 10 | 9.67 |
| 10.0 | 120 | 8 | 10 | 10 | 13.56 |

^a^Free [Mg^2+^]_i_ was calculated using WebMaxC software.

**Suppl. Table S2.** Internal solutions used to study the effects of free [Ba^2+^]_i_ on TRPM7 currents.

| ^a^Free [Ba^2+^]_i_  (mM) | Cs-glutamate  (mM) | NaCl  (mM) | Cs-HEPES  (mM) | Cs-EGTA  (mM) | BaCl_2_  (mM) |
| --- | --- | --- | --- | --- | --- |
| 0.55 | 120 | 8 | 10 | 10 | 9.55 |
| 1.00 | 120 | 8 | 10 | 10 | 10.49 |

^a^Free [Ba^2+^]_i_ was calculated using WebMaxC software.

**Suppl. Table S3.** Internal solutions used to determine Mg·ATP concentration-dependent inhibition of TRPM7 currents.

| ^a^[Mg·ATP]_i_  (mM) | Free ^b^[Mg^2+^]_i_  (µM) | Cs-glutam.  (mM) | NaCl  (mM) | Cs-HEPES  (mM) | Cs-EGTA  (mM) | Cs-EDTA  (mM) | Mg·ATP  (mM) | MgCl_2_  (mM) |
| --- | --- | --- | --- | --- | --- | --- | --- | --- |
| 0 | 250 | 140 | 8 | 10 | 10 | 3 | 0 | 0.38 |
| 0.4 | 250 | 120 | 8 | 10 | 10 | 3 | 0.57 | 3.19 |
| 1.5 | 250 | 120 | 8 | 10 | 10 | 3 | 2.15 | 2.71 |
| 3 | 250 | 120 | 8 | 10 | 10 | 3 | 4.30 | 2.10 |
| 4 | 250 | 120 | 8 | 10 | 10 | 3 | 5.70 | 1.70 |
| 5 | 250 | 120 | 8 | 10 | 10 | 3 | 7.10 | 1.28 |
| 6 | 250 | 120 | 8 | 10 | 10 | 3 | 8.55 | 0.85 |
| 9 | 250 | 120 | 8 | 10 | 10 | 3 | 12.4 | - |

^a,b^[Mg·ATP]_i_ and free [Mg^2+^]_i_ were calculated using WebMaxC software.

**Suppl. Table S4.** Internal solution containing of 9 mM [Mg·ATP]_i_, and 0.55 or 1 mM [Mg^2+^]_i_.

| ^a^[Mg·ATP]_i_  (mM) | Free ^b^[Mg^2+^]_i_  (µM) | Cs-glutam.  (mM) | NaCl  (mM) | Cs-HEPES  (mM) | Cs-EGTA  (mM) | Cs-EDTA  (mM) | Mg·ATP  (mM) | MgCl_2_  (mM) |
| --- | --- | --- | --- | --- | --- | --- | --- | --- |
| 9 | 550 | 140 | 8 | 10 | 10 | 3 | 10.7 | 2.10 |
| 9 | 1.00 | 140 | 8 | 10 | 10 | 3 | 9.9 | 3.55 |

^a, b^[Mg·ATP]_i_ and free [Mg^2+^]_i_ were calculated using WebMaxC software.

**Suppl. Table S5.** Biophysical properties of the WT and N1097Q channel variants obtained in outside-out recordings.

|  | 0 mM [Mg^2+^]_i_ | | 1 mM [Mg^2+^]_i_ |
| --- | --- | --- | --- |
|  | WT (*n* = 7) | N1097Q (*n* = 7) | N1097Q (*n* = 7) |
| *N* | 2 - 6 | 1 - 6 | 2 - 5 |
| *i* (pA) | -2.2 ± 0.21 | -1.3 ± 0.13* | -1.2 ± 0.09 |
| *γ_C_* (pS) | 36.1 ± 3.46 | 20.6 ± 0.79* | 20.5 ± 1.08 |
| *NP_O_* | 0.63 ± 0.112 | 0.58 ± 0.156 | 0.61 ± 0.117 |
| *T_OS_* (ms) | 4.7 ± 0.63 | 1.5 ± 0.30 * | 1.6 ± 0.29 |

*N*: number of channels being simultaneously active in a given patch;

*i*: single channel amplitude at -60 mV;

*γ_C_*: single channel chord conductance at -60 mV (assuming a reversal potential of 0 mV in symmetrical Na^+^);

*NP_O_*: probability of being open simultaneously of the *N*^th^ channel;

*T_OS_*: surrogate mean open time (product of *NP_O_* and total recording time divided by the number of openings);

*n*: number of independent cells measured;

*: significant difference from WT.

**Table S6.** Interactions of residues 1097 and 1098 stabilizing the closed gate in simulated TRPM7 channels.

| Simulated system | The average number of inter-subunit hydrogen bonds | | | Number of  Residue 1097-Mg^2+^ interactions |
| --- | --- | --- | --- | --- |
|  | Res1097-Res1097 | Res1097-Res1098 | Total |  |
| WT (closed) | 1.61 | 2 | 3.61 | - |
| WT-MG | 0.17 | 1.94 | 2.11 | 4 |
| WT (open) | 0.09 | 0.27 | 0.36 | - |
| N1097A | 0 | 3.23 | 3.23 | - |
| N1097Q | 1.39 | 0.84 | 2.23 | - |
| N1097-MG | 1.81 | 1.42 | 3.23 | 1 |
| N1098A | 0.03 | 0 | 0.03 | - |
| N1098D | 0.1 | 0 | 0.1 | - |
| N1098E | 0.65 | 0.62 | 1.27 | - |
| N1098Q | 0.39 | 0.23 | 0.62 | - |
| N1098Q-MG | 0.83 | 0.72 | 1.55 | 4 |
